# Reading the Social Clock: Analyzing Nonverbal Coordination Dynamics in Casual Chat and Conflict

**DOI:** 10.1111/nyas.70118

**Published:** 2025-10-26

**Authors:** Gary Bente, Ralf Schmälzle, Nolan Jahn, Mark Reimers

**Affiliations:** ^1^ Department of Communication Michigan State University East Lansing Michigan USA; ^2^ Institute for Quantitative Health Sciences and Engineering Michigan State University East Lansing Michigan USA

**Keywords:** alignment, motion capture, movement coordination, nonverbal communication, synchrony

## Abstract

While temporal coordination of nonverbal behaviors is considered crucial for successful communication, the underlying behavioral phenomena are poorly understood, and it remains unclear how coordination dynamics adapt to different social task demands. This study explores changes in interpersonal synchrony as dyads transition from a casual getting‐acquainted conversation to a hierarchical conflict resolution task. Using full‐body motion capture, we compared temporal alignment patterns in movement activity, head orientation, and body posture of 66 dyads interacting in both conditions. Results show that different coordination measures responded differentially to the situational demands. Time‐based measures reveal changes in movement coordination in most body dimensions when transitioning to conflict. Frequency‐based measures show differences in the higher frequency bands (1–2 Hz) for horizontal and vertical head motions. Correlations between conditions were found in the spatial domain, indicating dyad‐specific, persistent levels of postural similarity. The results suggest a nuanced understanding of temporal coordination phenomena, highlighting the need to consider different nonverbal subsystems and distinct types of coordination.

## Graphical abstract

This study explores changes in interpersonal coordination as dyads transition from a casual chat to conflict. Multiple measures of movement coordination were derived from full‐body motion capture data collected from 66 dyads. Observer ratings were based on computer animations of neutral characters. Results show that different coordination measures responded differentially to the situational demands. No systematic correlations were found between behavioral measures, observer ratings, and self‐reports of interaction quality.

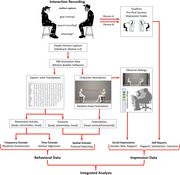

## 1 Introduction

Defining *chronemics* as a pivotal domain within communication research, Bruneau [1] asserts: “Time is intricately related to how people talk, the nature of communication environments, and the nature of communication events which mediate and sustain social order and organization. Time may be the basis of all nonverbal communication” (p. 108). Accordingly, the utilization of time must be regarded as a vital element in nonverbal communication, indispensable for coordinated action and collaboration, and essential for the coconstruction of meaning in social encounters [2].

An important distinction in the study of nonverbal timing phenomena lies in the focal point of analysis, delineating either the individual or the dyad as the fundamental unit of observation [3]. Observations at the individual level encompass timing features that can be assessed independently of an interaction partner, such as the tempo, velocity, or fluency of movements. These features are supposed to inform inferences about intrapersonal processes, such as intentions and emotions [4, 5, 6, 7, 8]. Conversely, observations at the dyad level pertain to temporal coordination phenomena that can only be evaluated relative to, or in conjunction with, the behavior of a counterpart. From a human ethology perspective, these phenomena have been conceptualized as a dynamic system of “cross‐modal attunement [in which] all elements continuously interact and evolve to form a mutual aggregate pattern known as coregulated activity” [9, p. 16]. This subtle orchestration, emerging at the system level, often referred to as *interpersonal synchrony* [10] or *interpersonal coordination* [11], is a remarkable aspect in human interactions transcending the exchange of individual nonverbal signals.^1^ It has been shown that the successful attunement of nonverbal behaviors not only facilitates a smooth flow of verbal exchange [12, 13], but also fosters relational qualities such as rapport, trust, and cooperative motivation [14, 15, 16, 17, 18]. The literature offers various explanations for why people coordinate their movements and find synchrony rewarding [14]. In most general terms, coordinated motor action creates synergies [19] that support joint goal achievement, thereby improving the group's survival chances [20, 21]. Cognitive accounts emphasize the energy optimization principle of the human brain, suggesting that behavior matching and synchrony lead to a “nearness of self and other representation” [22], reducing the complexity of the social environment [22, 23], and allowing for less effortful prediction of others’ behaviors [24]. Compatible with the cognitive account, Bente and Novotny [14] highlighted the potential *diagnostic value* of interpersonal synchrony. They argue that synchrony achieved during initial casual conversations “…might indicate the ease or difficulty in coordinating actions with a respective partner later, when facing collaborative task demands […] which identifies the other as a potential collaborator or even life partner when things become serious” (p. 419). This idea echoes earlier research in human ethology that emphasized the role of nonverbal attunement in courtship behavior. In that context, Grammer et al. [25] described synchronization as “a sign of mutual understanding in interactions (that is, the higher the degree of synchronization, independent of the type of synchronization, the higher the degree of mutual understanding). The amount of synchronization achieved in an interaction would thus be an indicator of compatibility between the interactants” (p. 8).

We contend that the diagnostic value of nonverbal synchrony extends beyond courtship interactions to a broader range of interpersonal situations characterized by uncertainty. This applies not only to initial encounters, where mutual “evaluation forces” seem to be particularly strong [26, p. 287], but also to situations where relationships are tested by disruption, argument, or conflict [27, 28, 29]. Notably, the role of nonverbal coordination/synchrony in conflict and the link between spontaneous nonverbal coordination in initial encounters and in contentious interactions has yet to be systematically studied. The current study addresses this gap by using a within‐dyad design to compare coordination patterns during initial getting‐acquainted conversations and a subsequent hierarchical conflict resolution task.

### 1.1 Nonverbal Coordination/Synchrony in Conflict

While there is evidence that interpersonal synchrony and coordination are closely associated with rapport [26], and that rapport is essential for cooperative conflict resolution [30], associations between behavioral coordination and conflict have been rarely studied directly and results are inconclusive. For instance, Drolet and Morris [31] emphasize the role of face‐to‐face contact for the establishment of rapport and demonstrate the mediational effect of initial rapport in the resolution of later conflict. In lack of behavioral data, though, the specific contribution of nonverbal coordination to conflict resolution remains unclear. Results of the few studies including behavioral measures of coordination in the study of argument or competitive debates are inconclusive. For instance, Paxton and Dale [28] found that in‐phase movement synchrony significantly decreased during arguments compared to affiliative settings. Conversely, Tschacher et al. [29] report increases in synchrony levels when debates were conducted in competitive as compared to a cooperative climate. Similarly, Lozza et al. [27] found that “…individuals significantly synchronized their movement during a competitive debate,” suggesting that “…nonverbal synchrony depends on task instructions and is particularly high in competitive settings” (p. 190).

These contradictory results leave open whether argument or conflict hinder behavioral synchrony or, conversely, enhance nonverbal coordination efforts to navigate relational challenges. This question is closely tied to the more general question whether interpersonal synchrony serves a functional purpose or is merely “an epiphenomenon—a functionless by‐product of another interpersonal process” [10, p. 2]. If temporal coordination is merely a symptom, that is, a reflection of a harmonious relationship or smooth interaction, it would likely decrease during conflict. However, if it is functional as a mitigation mechanism, coordination levels could also increase during conflict—provided both parties have a strong motivation in preserving the relationship and resolving the conflict collaboratively. In lack of this motivation the conflict could result in communication or relationship rupture.

A study by Deres‐Cohen et al. [32] supports this view. Analyzing nonverbal synchrony as a potential marker of alliance ruptures in psychotherapy, they found, that synchrony was associated with “confrontational ruptures” but not with “withdrawal ruptures.” Evidently, the desire to maintain the relationship, even under difficult conditions, invoked higher levels of nonverbal entrainment, while withdrawal tendencies were associated with desynchronized behavior (for a recent review on the role of interpersonal coordination in therapeutic alliance ruptures, see [33]). It remains an open question though if these results also apply to nontherapeutic, mundane interactions.

The current study specifically investigates: (1) whether and how interpersonal coordination dynamics change as a dyad transitions from a casual conversation to a hierarchical conflict; (2) whether initial dyadic coordination covaries with behavioral coordination during the subsequent conflict; (3) how interaction outcomes—such as perceived rapport, communication satisfaction, and conflict resolution—covary with the level of nonverbal coordination achieved; and (4) how different measures of synchrony and coordination converge or diverge in their explanatory value with respect to interaction outcomes and situational variance.

### 1.2 Facets of Interpersonal Synchrony/Coordination

Current literature offers multiple definitions and measures of behavioral synchrony/coordination [34], leaving it unclear whether identical labels are being used for different phenomena or different labels for the same underlying processes [35]. Narrow definitions of synchrony—as the rhythmic entrainment of weakly coupled oscillators [14]—are certainly appropriate for the coordination of unimodal, inherently rhythmic motor activities such as clapping [36], tapping [37], waving [38], walking in stride [39], or rocking in chairs [40]. However, it remains questionable whether such a definition adequately captures the more complex and multimodal coordination dynamics involved in spontaneous social interactions and conversations. Nonverbal behavior during conversations is typically less rhythmic, orchestrated across multiple channels—including gaze, gestures, posture, and body movements—and tightly interwoven with verbal exchange. Moreover, the form and degree of interpersonal coordination can fluctuate as conversations evolve, relationships develop, socioemotional demands shift, or contextual factors change [10]. Given these complexities, it may be advisable to abandon the use of *synchrony* as an umbrella term in conversational research and instead adopt the broader construct of interpersonal coordination, which better accommodates the multimodal, context‐sensitive, and often nonrhythmic nature of nonverbal behavior. Synchrony may still be applicable to specific facets of coordination, particularly where rhythmic properties can be theoretically expected or empirically demonstrated in individual nonverbal subsystems, such as head nods, shakes, or beat gestures.

This study seeks to address the complexity of nonverbal coordination dynamics in two ways, first by distinguishing between movement channels that differ in their temporal characteristics and communicative functions, and second by differentiating between facets of synchrony/coordination as defined by the respective analytical methods employed [10, 15, 35, 41, 42]. With respect to motion dimensions, the analysis considers nonverbal subsystems—namely, the movements and postures of the body, head, upper, and lower extremities. Regarding the facets of synchrony and coordination, the study adopts operational definitions embedded within the specific analysis methods. It incorporates three analytical perspectives: (1) frequency‐domain analyses that capture rhythmic entrainment of movement, (2) time‐domain analyses that assess the temporal alignment of activity levels, and (3) spatial‐domain analyses that examine similarity in postural configurations and spatial orientation.

The differentiation between frequency‐based and time‐based approaches refers to the assumption of *periodicity* (see [34]). While periodicity might not be as evident in conversations compared to genuinely rhythmic movements (clapping, marching), rhythmic patterns can be hidden in the mesh of the multiple hand gestures, head and body movements occurring in spontaneous interactions [43]. In this sense, frequency‐based approaches, focusing on the *rhythmic entrainment* of certain motion dimensions might come closest to the narrower definition of synchrony [14]. A prominent method to detect rhythmic entrainment patterns in social interactions in various frequency bands is wavelet transform coherence (WTC, [44]).

Time‐based analysis methods do not assume periodicity in motion signals. Instead, they aim to quantify the covariation of activity patterns over time, typically using cross‐correlation techniques [45, 46]. The term *temporal alignment* may more accurately reflect the coordination phenomena captured by these methods than the term synchrony. Notably, both frequency‐ and time‐based approaches acknowledge that entrainment or alignment may involve temporal shifts between individual time series and are therefore designed to account for time lags.

Whereas frequency‐ and time‐domain approaches to coordination typically abstract from formal features of nonverbal behavior, such as bodily configurations and spatial alignment, other frameworks—centered on concepts like mimicry, mirroring, or behavioral matching—emphasize the *spatial dimension* of coordination. These approaches highlight the importance of morphological similarity, focusing on spatial features such as posture and body orientation as key indicators of interpersonal alignment [47, 48, 49, 50]. Although synchrony and behavioral matching are often treated as conceptually distinct [51], the effectiveness of copying behavior has been shown to depend critically on temporal proximity: the mirrored behavior must follow the original action within a short time window—but not simultaneously—in order to be perceived as socially meaningful [52]. The current study includes spatial pattern matching as a further facet of interpersonal coordination, complementary to coordination phenomena in the time and frequency domains.

### 1.3 Motion Capture as Nonverbal Ground Truth

The described variety of nonverbal coordination phenomena, and specifically the differentiation of bodily dimensions (e.g., head and body movements, hand gestures) as well as their dynamic features (postures, movements, orientations) place significant demands on the data collection and the spatial resolution of the nonverbal raw data. It is important to note that commonly used video‐based methods for data acquisition, such as motion energy analysis (MEA, [53]), abstracting from the complexity construed by nonverbal subsystem and motion features may fall short of capturing the required level of detail. We therefore opt for the use of motion capture technology, which provides detailed translation and rotation data for all joints of the human body, thus allowing for the analysis of motion aggregates for separate body regions, as well as the determination of spatial orientation patterns and postural similarities [54, 55, 56, 57]. Furthermore, another benefit of motion capture data is that they can be used for virtual character animations, which provides a powerful method for stimulus creation in person perception studies [58, 59]. The current study draws upon this option in the collection of observer ratings using neutral virtual characters to mask aspects of physical appearance potentially relevant to stereotype influence (for instance gender, age, race).

### 1.4 The Current Study

**FIGURE 1:**
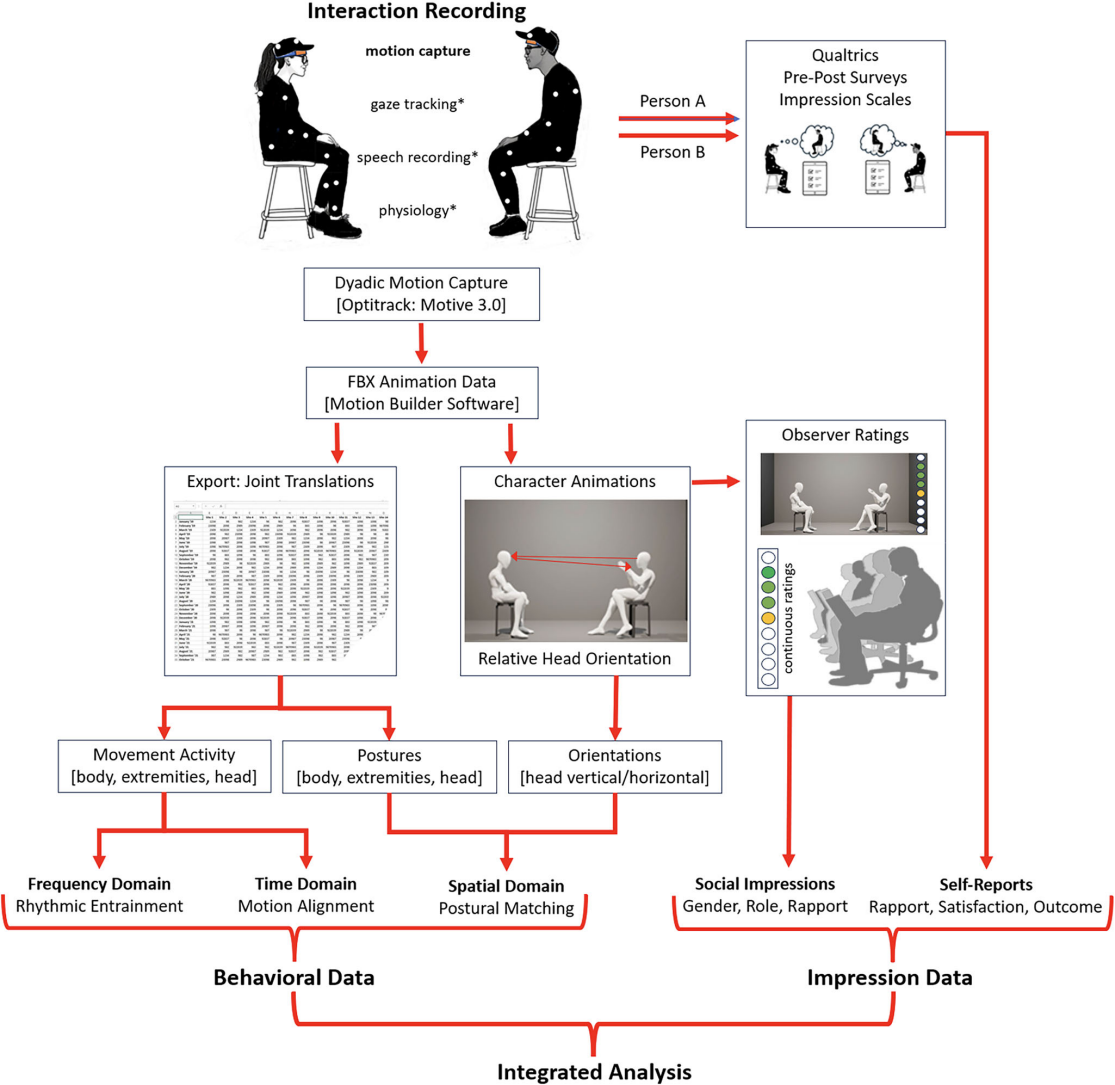
Study setup, timeline, and analysis paths.

The current study adopts a multimodal, multimethod approach, encompassing interpersonal coordination dynamics across different nonverbal subsystems (body, head, lower and upper extremities), features (motion, orientation, body posture), and coordination mechanisms observable either in the frequency or time domain or in the spatial domain. Conceiving of the dyad as the smallest unit of analysis the study pursues a strict within‐dyad, two‐sided analysis approach to test possible increases and decreases in the coordination levels of dyads transitioning from a casual getting‐acquainted conversation to a hierarchical conflict. Full‐body motion capture serves as the major data source. Observer ratings of rapport as well as interactants’ self‐reports of perceived rapport, interaction satisfaction, and outcome are included as subjective data elements. Figure 1 gives an overview over the study design, and the data collection and analysis pipelines. It has to be noted that data collection encompassed several variables that are reported in a different context. These include speech recordings, eye tracking, and physiological measures as well as a broader set of self‐report variables including personality surveys, impression ratings, and emotion judgments. The different steps of data collection and analysis displayed in the schematic are explained in detail in the method section which repeatedly refers to this figure.

To that end, this study addresses four primary research questions. First, we investigate whether and how interpersonal coordination dynamics change as dyads transition from a casual conversation to a hierarchical conflict. Second, we examine whether a dyad's initial coordination patterns covary with their coordination during the subsequent conflict. Third, we explore how interaction outcomes—such as perceived rapport, satisfaction, and conflict resolution—relate to the level of nonverbal coordination achieved. Finally, by employing a variety of analytical techniques, we assess how different measures of coordination converge or diverge in their explanatory power regarding situational changes and interaction outcomes.

## 2 Method

### 2.1 Participants

In total, 132 participants (66 dyads; 78 self‐identified female, 49 self‐identified male; 27 female–female, 10 male–male, and 29 mixed–gender dyads) participated in the study. Participants average age was 19.95 years (SD = 2.95). The study was approved by the local IRB. Participants were recruited via word‐of‐mouth propaganda and local SONA participant systems. They provided written informed consent to the protocol, including the sharing of anonymized data. Participants received an incentive of $20 for about an hour of their time.

### 2.2 Procedures

All interaction recordings took place in the motion capture lab. Participants, who were unacquainted, entered the motion capture facility through two separate doors into two different sections of the lab divided by a curtain, so they could not meet each other before the first interaction session. Upon arrival, they provided written consent, filled out a presurvey on an iPad, and watched an explainer video about the measures and devices. They were left alone instructed how to put the motion capture data suit on top of their clothing. When ready, the experimenter attached the markers to the data suits and helped to put on the Muse 2 head band for the physio data collection as well as the eye tracking glasses and mobile recorders also used for speech recordings.^2^ Once all sensors were attached, the participants were led into the motion capture room and seated on two stools placed 6 feet apart. They were then told to take off their face masks (Covid‐19 mandate) and given the instructions for the get‐to‐know (GTK) task, which basically stated that they would have 5 min to GTK each other. The task was started with a clap from the experimenter, which also served as the synchronization signal for the different recordings. Participants were given a signal after 5 min to end the GTK task and they were handed iPads to complete surveys about their experiences and impressions during the interaction (see Section 2.3 for details). The iPads were returned to the experimenter after completion of the survey before starting the second interaction.

The participants were then handed laminated instruction sheets for the managerial task [58, 59]. The managerial task instruction aimed to introduce power and conflict into the interaction by assigning one participant to the role of the boss of a fictional company and the other participant to the role of the employee. The role of the boss was always assigned to the right chair, but the assignment to the chairs was random. The instruction stated that a disciplinary feedback conversation was about to take place. The context given to the boss was that there were reports and evidence of the employee's insubordination (e.g., deliberate absence from meetings and reticent behavior), but at the same time, the employee brought in the strongest sales numbers and thus should not be lost as an employee. For the employee, the instructions also emphasized the same parameters, that is, a mix of strong performance but incompatible team behavior. In sum, these instructions served to create a power imbalance and a mixed motivation, that is, both participants want to achieve their goals. Once both participants signaled readiness, instruction sheets were returned to the experimenter and the conflict interaction was started upon the clap‐signal, again serving as marker for data synchronization. As for the GTK interaction, the conflict interaction was terminated by the experimenter after 5 min. Several conflict interactions were terminated by the participants before the time expired. For the behavioral coordination analysis, we used the first 3 min of each interaction or the full interaction if shorter. When the interaction came to an end, participants were again handed iPads to complete surveys about their experiences and impressions during the conflict interaction (see Section 2.3 for details).

### 2.3 Measures

#### 2.3.1 Behavioral Measures

Participants movement activity was recorded simultaneously with an Optitrack 12‐camera (Prime 16) marker‐based motion capture system. Interactants wore black data suits (available in three sizes) with 40 reflective markers attached (see Figure 1). Movement data were recorded at 120 Hz using the capture software Motive 3.0 (NaturalPoint). The software also recorded the sound including a start signal (experimenter's hand clap) that was shared by all recording channels (see Figure 1) to be used for later data synchronization.

As visualized in Figure 1, the mocap data were exported from the capture software to a standard animation format (.fbx) containing translations and rotations of all skeletal joints. A custom Python plugin was used to import the .fbx files into the animation program Motion Builder (Autodesk) and to export the global translation and rotation information (world coordinates) of the joints into easily readable, tab‐delimited ASCII .csv files with a rate 25 frames per second (fps). The joint translation data were used as major input for the analysis in the spatial domain. Another custom Python plugin was used to automatically characterize the skeleton information within Motion Builder and to create neutral character animations of all interactions. These animations served as stimuli in the observer study (see below) and were also used to derive two further spatial parameters, that is, the vertical and horizontal head orientation relative to the partner head center. The rationale of the software program using a ray‐tracing algorithm realized in Vizard 3.0 Python is detailed in Supporting Information 1. The program issues the deviation of a virtual pointer placed on the animated character's forehead from the direct line of view (line between the midpoints of both foreheads).

While joint translations and head orientations formed the basis for spatial analysis, the time‐ and frequency‐based analyses were based on motion data. For each joint motion was calculated as Euclidian distance of the consecutive time points’ translation vectors. Motion time series of the respective joints were then aggregated to represent the activity of six subsystems dimensions: whole body, trunk, head, upper extremities, lower extremities. Head motion was further detailed using the dynamic changes in the horizontal and vertical head orientation, resulting in an overall set of eight nested movement components. The same aggregation rationale was used for spatial analysis also referring to the same eight subsystems (see below). All analyses were based on the frame rate of 25 fps as exported from MotionBuilder. The motion time series extracted for the eight body dimensions was further preprocessed applying a Butterworth low pass filter with a filter constant of 2 Hz to reduce high frequency jitter.

Analyses in the frequency domain were based on WTC realized in MATLAB [43]. The WTC used here is the normalized version of the wavelet cross‐spectral density, providing a bounded measure (from 0 to 1) of the degree of correlation between two time series for selected frequency bands. Put simply, wavelet–coherence measures how strongly two signals are related to each other over time and across different frequencies. It shows when and at what frequency the patterns in the two signals match or fluctuate in phase. Statistical analysis focused on averaged coherence scores in three frequency bands: 0.25–0.5 Hz, 0.5–1.0 Hz, and 1.0–2.0 Hz.

Analyses in the time domain were based on rolling‐window time‐lagged cross correlations (RWTLCCs, Python code adapted from [60]. RWTLCC calculations were performed in steps of two frames (0.2 s) along the time axis and across time lags ranging from −5 to +5 s. Each correlation was computed within a moving window of 10 s. Fisher z‐transformation was applied to all cross‐correlation values prior to further analysis. For statistical evaluation, the resulting data matrices (observation time × time lag) were aggregated along the time axis, yielding a one‐dimensional array of average correlations at each lag. From this array, two measures were extracted for further analysis: (1) the *peak correlation*—the highest average *r* value across all lags, and (2) the *peak correlation lag*—the time lag (in seconds) at which this maximum occurred.

Postural similarity was calculated as differences of both interactants’ joint translations using a custom Python routine [51, 54]. A detailed description of the procedure can be found in Supporting Information 2. Figuratively speaking, both skeletons were superimposed by replacing the bodies in the same origin (relative to the individual root joint translation, i.e., the hip) and rotating one of the interactants body by 180°. Euclidian distances were then calculated for all corresponding joints resulting in a similarity matrix (time × joints) for each dyad. The activity of the singular joints was then aggregated across the joints belonging to one of the subsystems resulting in activity time series for whole body, trunk, head, upper and lower extremities. The mean of distances for each subdimension over time was used as parameter for further analyses.

Spatial orientation similarity in the vertical and horizontal head dimension were calculated in a different way. As both time series already contained one‐dimensional deviation measures relative to the partner (see Supporting Information) we applied windowed time‐lagged cross correlations (RWTLCC) to the inherent spatial information as we did for the dynamic variations (first derivate of the orientation time series) before. Unsigned values were used for the horizontal head deviation, ignoring if the deviation was to the right or the left. Vertical head deviation was analyzed based on the signed values (up or down from the direct line of view).

#### 2.3.2 Self‐Report Measures

The analysis of self‐report measures focuses on three dimensions: perceived rapport, satisfaction with the outcome, and a combined score of the interaction quality scale (cf. [61]). These measures, obtained after both the GTK session and the managerial conflict (MAN) interaction, form part of a broader spectrum of self‐report data collected throughout the study.^3^ Specifically, perceived rapport was rated by each interactant after each interaction using a 7‐point scale in response to the question “We had good rapport” (strongly disagree, 1 to strongly agree, 7). Likewise, satisfaction with the outcome of the interaction was measured in response to the question “I was satisfied with the outcome of the interaction” (7‐point scale). Finally, we adopted the interaction quality scale from [61]. This scale originally comprises 18 items focusing on interaction qualities like “the interaction was boring,” “the interaction had focus,” “the interaction was friendly” (see Supporting Information). One item was dropped due to low interitem correlations, resulting in a 17‐item scale (7‐point scales) with very good reliability, Cronbach's α = 0.89

#### 2.3.3 Observer Ratings

**FIGURE 2:**
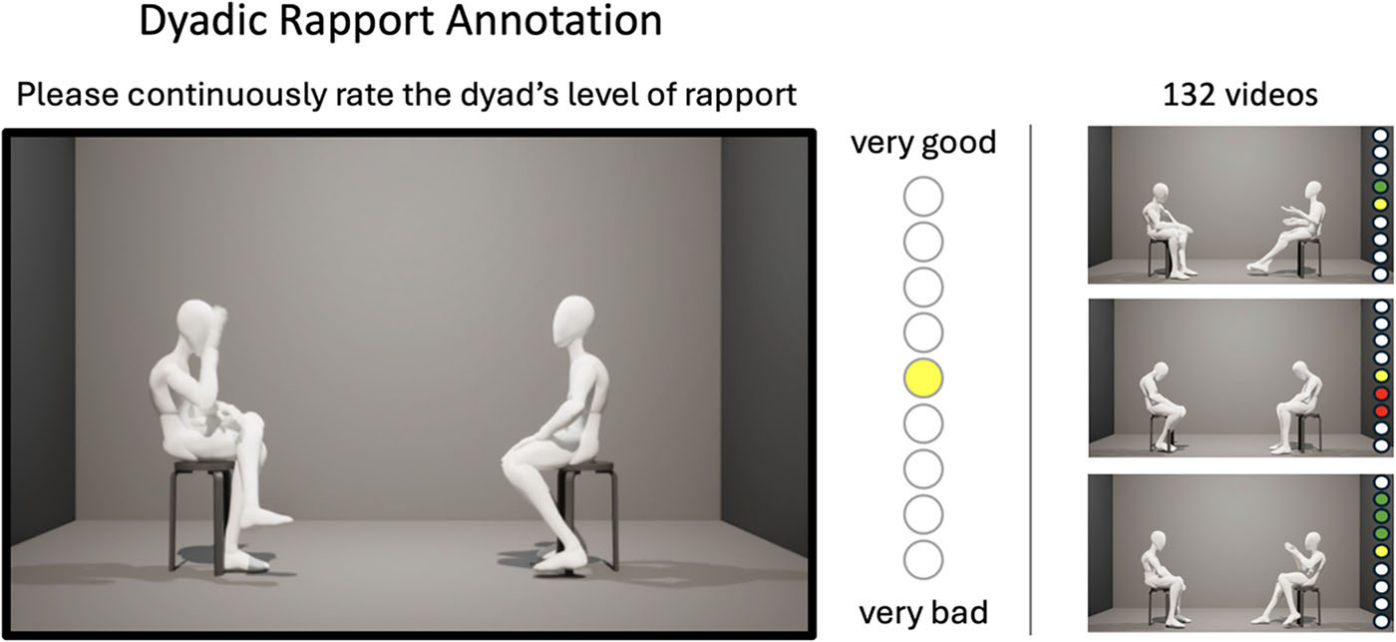
Annotation stimulus and program interface for the continuous rapport rating. Screenshot showing the avatar animation and the 9‐point continuous response measure (CRM) scale. Participants annotated the level of rapport deemed to be between the interactants by adjusting the up and down keys on a keyboard.

To avoid influences of verbal content as well physical appearance features of the interactants, observer ratings were based on character animations featuring the same neutral avatar for all interaction participants. Character animations were produced using a custom Python plugin within MotionBuilder (see Figure 1). The program generates the animations for the whole dataset (*N* = 132) in one loop requiring no manual action. Details of the software routine can be found in Supporting Information 4. An example of the output is shown in Figure 2.

Six coders were recruited by word of mouth from the undergraduate student population of the Department of Communication at Michigan State University. For overall 5 h of work, they received an incentive of US$120 each. Coding was performed individually by all 6 coders using a custom Python program, developed within the Vizard 3.0 VR platform. The VR application allows you to play prerendered .mp4 files as well as to perform real‐time animations of the .fbx files. The coding procedure comprised two parts. Part 1 required the coders to continuously rate the level of rapport during the first minute of each interaction using a 9‐point slider scale displayed at the right side of the animation (see Figure 2). Coders were instructed according to previous practice (see [54]). The Python program showed the interaction sequences in random order. Each coder had the same number of original and flipped stimuli (see section stimuli) and all stimuli were equally often presented in the original and the flipped version. Part 2 of the coding procedure was administered after the continuous rapport ratings. Video stopped and questions and rating items were displayed on a canvas in the 3D environment. The coders then answered questions about the assumed gender and role (boss or employee) of each interactant and rated likability, activity, and dominance of both interactants on 7‐point rating scales. The following analysis focuses on the rapport ratings while results of the post hoc judgments will be reported in a different publication.

## 3 Results

### 3.1 Results for the Behavioral Measures

#### 3.1.1 Entrainment of Movement Rhythms (Frequency Domain)

**FIGURE 3:**
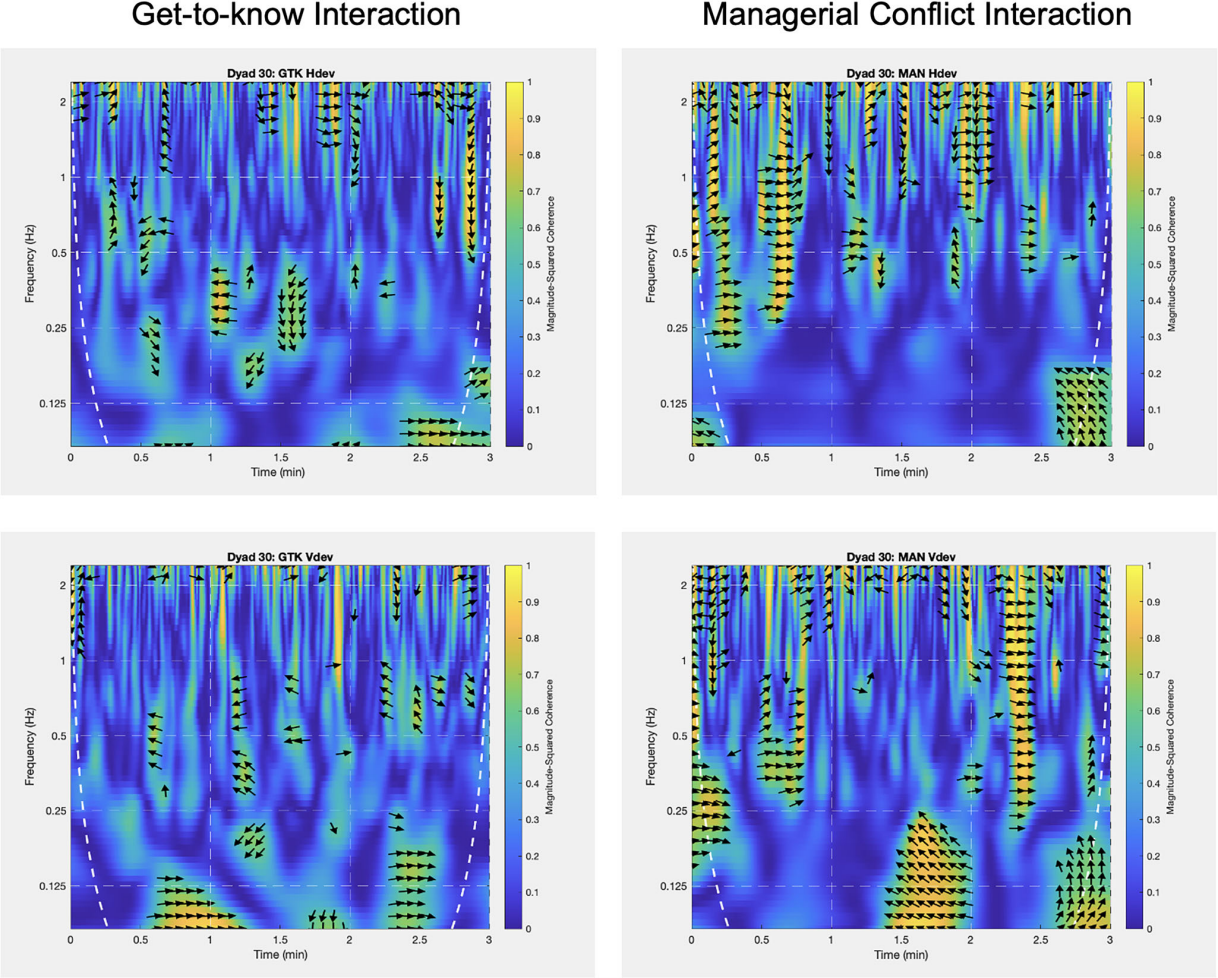
Wavelet–coherence patterns for one dyad, which show the differences in the horizontal head motion in the upper row and the vertical head motion in the lower row between the get‐to‐know (GTK) interaction in the left column and the managerial conflict (MAN) interaction in the right column, demonstrating the significant differences in the 1–2 Hz frequency. The white dotted line marks the cone of influence (COI).

Table 1 shows the results of paired *t*‐test comparisons (*p* values FDR corrected for *p* = 0.05) between initial conversations and conflict interactions for the coherence averages in three frequency bands for each of the eight motion subsystems (whole body, trunk, upper extremities, lower extremities, and head, as well as horizontal and vertical head movements). Differences in motion synchrony between the two interaction conditions could only be found in the 1–2 Hz frequency band for both the vertical and the horizontal head motion, indicating that faster oscillations in these dimensions showed larger coherence in the managerial as compared to the GTK. Table 1 also shows that with one exception (trunk motion), none of the coherence values showed significant correlations between the interaction tasks. Figure 3 shows the wavelet–coherence plot for the vertical head motion for an exemplary dyad demonstrating higher coherence levels in the 1–2 Hz frequency band in the GTK interaction as compared to conflict.

**TABLE 1:** Paired *t*‐test comparisons (FDR corrected) of standardized coherence scores in three frequency bands for seven body dimensions (wavelet transform coherence, or WTC).

	Means (SDs)		Paired *t*‐test			
**Variables**	**Initial (GTK)**	**Conflict (MAN)**	***t*(65)**	***p***	**Cohen's *d***	**Pearson *r***
**Whole body (aggregate)**						
1.0–2.0 Hz	0.259 (0.02)	0.260 (0.025)	−0.455	0.725	−0.056	0.145
0.5–1.0 Hz	0.229 (0.023)	0.224 (0.029)	0.941	0.491	0.116	−0.016
0.25–0.5 Hz	0.245 (0.034)	0.236 (0.041)	1.376	0.364	0.169	−0.004
**Trunk (aggregate)**						
1.0–2.0 Hz	0.268 (0.027)	0.268 (0.035)	0.137	0.891	0.017	**0.587**
0.5–1.0 Hz	0.239 (0.029)	0.23 (0.028)	1.784	0.237	0.22	−0.106
0.25–0.5 Hz	0.254 (0.039)	0.241 (0.04)	1.932	0.237	0.238	0.075
**Upper extremities (aggregate)**						
1.0–2.0 Hz	0.247 (0.023)	0.252 (0.026)	−1.3	0.378	−0.16	0.249
0.5–1.0 Hz	0.212 (0.025)	0.214 (0.032)	−0.4	0.725	−0.049	−0.045
0.25–0.5 Hz	0.225 (0.041)	0.219 (0.043)	0.884	0.499	0.109	0.091
**Lower extremities (aggregate)**						
1.0–2.0 Hz	0.252 (0.023)	0.261 (0.035)	−1.842	0.237	−0.227	0.219
0.5–1.0 Hz	0.231 (0.026)	0.226 (0.032)	0.941	0.491	0.116	−0.079
0.25–0.5 Hz	0.248 (0.036)	0.245 (0.044)	0.449	0.725	0.055	0.007
**Head (aggregate)**						
1.0–2.0 Hz	0.268 (0.022)	0.264 (0.022)	0.981	0.491	0.121	−0.002
0.5–1.0 Hz	0.242 (0.022)	0.233 (0.031)	1.975	0.237	0.243	0.121
0.25–0.5 Hz	0.261 (0.036)	0.251 (0.046)	1.618	0.258	0.199	0.116
**Head (horizontal)**						
1.0–2.0 Hz	0.273 (0.031)	0.300 (0.043)	**−4.912**	**< 0.001**	−0.605	0.29
0.5–1.0 Hz	0.235 (0.029)	0.252 (0.047)	−2.634	0.074	−0.324	0.166
0.25–0.5 Hz	0.237 (0.032)	0.234 (0.051)	0.423	0.725	0.052	0.126
**Head (vertical)**						
1.0–2.0 Hz	0.275 (0.025)	0.297 (0.050)	**−3.222**	**0.021**	−0.397	0.089
0.5–1.0 Hz	0.248 (0.030)	0.256 (0.044)	−1.233	0.389	−0.152	0.079
0.25–0.5 Hz	0.249 (0.035)	0.260 (0.043)	−1.618	0.258	−0.199	0.011

Bold values represent significant effects.

Abbreviations: GTK, get to know; MAN, managerial conflict.

#### 3.1.2 Alignment of Movement Activity (Time Domain)

Table 2 shows the results of the *t*‐test comparisons (*p* values FDR corrected for *p* = 0.05) for both RWTLCC results for the eight motion subsystems. Consistent significant differences between the two situations were found for the peak correlations of head and trunk motion, showing higher peak correlations in the initial GTK interactions. Peak correlations for the horizontal head movements showed an inverse effect being higher in the conflict interactions. Peak correlation offset differed significantly for the movement activity in the whole body, the trunk, the lower extremities and the head, being significantly larger in the conflict interactions, with average lag differences between 0.4 and 0.8 s between conditions. Overall, alignment was more immediate in the GTK interaction (shorter lags to reach correlation maximum) compared to conflict. With one exception (horizontal head movement) dyads also achieved higher correlation peak level in the GTK chat than in the conflict interaction. Horizontal head motion (turning away from or toward the partner) however, (putatively a correlate of attention regulation) demonstrated higher peak correlation in conflict, Two parameters that reveal significant situational differences also showed significant correlations: the peak correlation of the horizontal head motion and the peak correlation lag of the trunk motion suggesting dyad‐specific alignment patterns that that were similarly modulated by the situation across all dyads.

**TABLE 2:** Paired *t*‐test comparisons (FDR corrected) of motion alignment for seven body dimensions (rolling‐window time‐lagged cross correlations, or RWTLCCs).

	Means (SD)		Paired *t*‐test			
**Variables**	**Initial (GTK)**	**Conflict (MAN)**	***t*(65)**	***p***	**Cohen's** ***d***	**Pearson** ***r***
**Whole body (aggregate)**						
Peak correlation	0.141 (0.049)	0.121 (0.068)	1.939	0.096	0.239	0.001
Peak correlation lag	1.97 (1.341)	2.694 (1.504)	**−3.24**	**0.006**	−0.399	0.202
**Trunk (aggregate)**						
Peak correlation	0.157 (0.056)	0.133 (0.066)	**2.556**	**0.03**	0.315	**0.246**
Peak correlation lag	1.476 (1.308)	2.188 (1.442)	**−3.663**	**0.005**	−0.451	0.353
**Upper extremities (aggregate)**						
Peak correlation	0.124 (0.043)	0.11 (0.059)	1.497	0.188	0.184	−0.067
Peak correlation lag	2.455 (1.449)	2.788 (1.481)	−1.361	0.208	−0.167	0.092
**Lower extremities (aggregate)**						
Peak correlation	0.142 (0.041)	0.139 (0.065)	0.375	0.709	0.046	−0.013
Peak correlation lag	1.624 (1.134)	2.473 (1.483)	**−3.921**	**< 0.001**	−0.483	0.132
**Head (aggregate)**						
Peak correlation	0.162 (0.049)	0.140 (0.063)	**2.397**	**0.038**	0.295	0.072
Peak correlation lag	1.352 (1.085)	2.112 (1.502)	**−3.354**	**0.005**	−0.413	0.028
**Head (horizontal)**						
Peak correlation	0.126 (0.061)	0.159 (0.079)	**−3.313**	**0.006**	−0.408	**0.384**
Peak correlation lag	1.967 (1.481)	1.418 (1.770)	1.898	0.096	0.234	−0.019
**Head (vertical)**						
Peak correlation	0.152 (0.041)	0.167 (0.079)	−1.463	0.188	−0.18	0.076
Peak correlation lag	0.942 (1.120)	1.155 (1.417)	−0.878	0.412	−0.108	−0.167

Bold values represent significant effects.

Abbreviations: GTK, get to know; MAN, managerial conflict.

**FIGURE 4:**
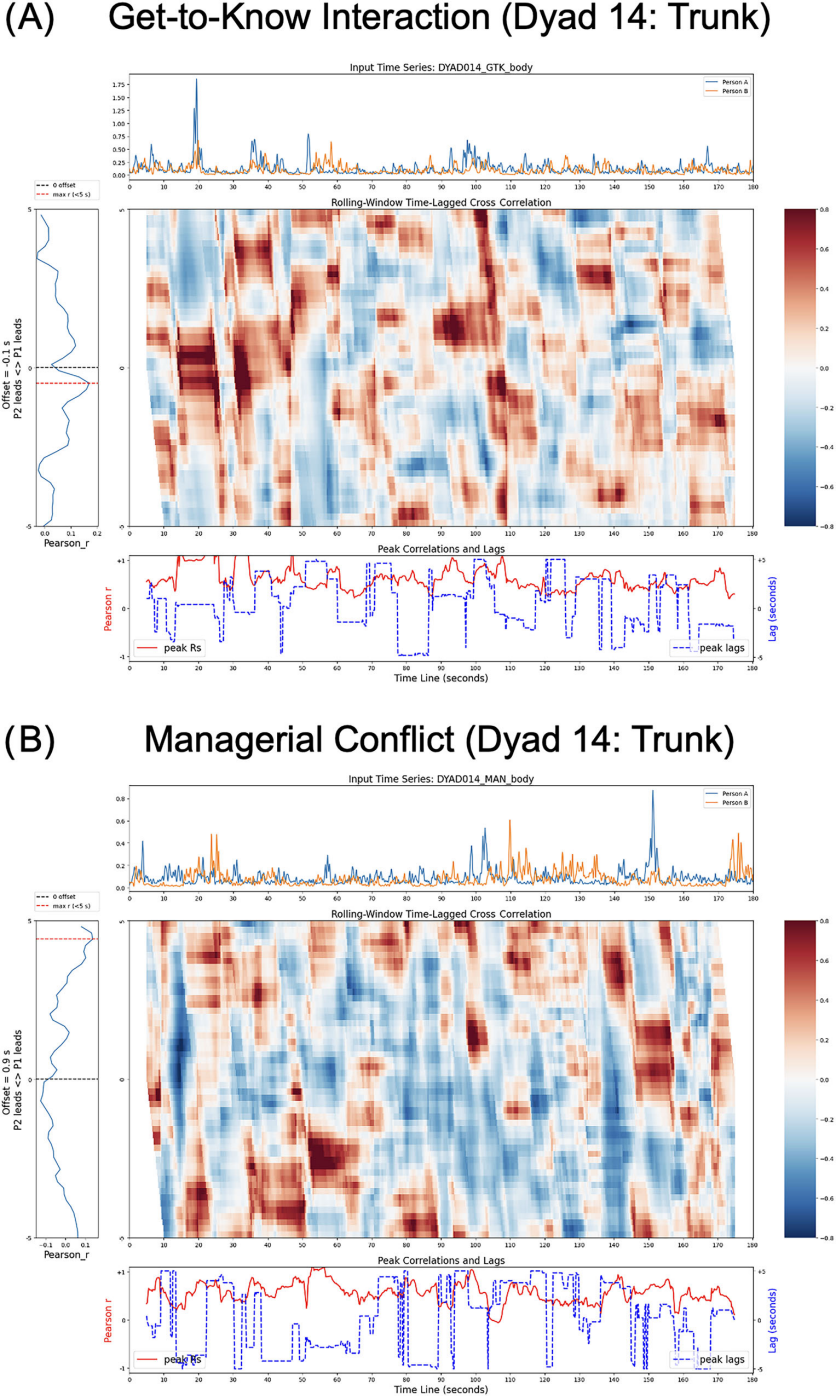
Visualization of rolling‐window time‐lagged cross correlations (RWTLCCs) results for the trunk motion of an exemplary dyad in (A) the get‐to‐know interaction and (B) the conflict interaction. Subplots within each panel: upper, motion time series of both actors; middle, cross correlations (Fisher z‐transformed) along the time lags (vertical axis) and the observation time (horizontal axis); left, average correlations for all time lags across the observation time showing optimal lag (red dotted line); and lower, maximal and average correlation at each time point across all lags.

Figure 4 illustrates the rationale of the RWTLCC analysis showing an example of temporal alignment differences between the conditions for the whole‐body motion in one dyad. The example specifically shows the difference in the peak correlation time lags visible in the center graph, with the highest correlations group much closer to the midline (zero lag) in the initial interaction as compared to the conflict interaction. This difference is also visualized in the left graph where the red horizontal line indicates the peak correlation lag based on the average correlation across time, in this case 1.65 s for the initial interaction and 3.15 s for the conflict interaction.

#### 3.1.3 Postural Matching: Interpersonal Joint Distances

Table 3 shows the results of the comparison of postural similarity achieved in six body dimensions for both interaction tasks. Following the procedure described above (see also Supporting Information), we averaged the sum of the Euclidian joint distances for each subsystem at each point across the observation time. Means and standard deviations served input for the paired *t*‐test. Except for the posture of the upper extremities (marginally significant) all mean values of the subsystems revealed significant differences with larger values in the conflict interaction indicating less postural similarity. The standard deviations of two subsystems (trunk and headjoints) also revealed significant differences pointing to larger variability in the postural matching during the conflict interactions. Interestingly, the means and standard deviations for all subsystems showed high, significant correlations between the two situations. indicating dyad‐specific levels of postural matching that was systematically modulated by the situational demands.

**TABLE 3:** Paired *t*‐test comparisons (FDR corrected) of postural similarity (aggregated Euclidean joint distances).

	Means (SD)		Paired *t*‐test			
**Variables**	**Initial (GTK)**	**Conflict (MAN)**	***t*(65)**	***p***	**Cohen's** ***d***	**Pearson** ***r***
**Whole body (aggregate)**						
Mean	178.65 (48.274)	194.571 (52.619)	**−3.505**	**0.005**	−0.431	**0.740**
SD	23.147 (11.821)	24.490 (10.484)	−1.11	0.339	−0.137	**0.623**
**Trunk (aggregate)**						
Mean	6.049 (2.773)	6.561 (2.665)	**−2.971**	**0.007**	−0.366	**0.870**
SD	1.174 (0.611)	1.372 (0.660)	**−3.177**	**0.006**	−0.391	**0.691**
**Upper extremities (aggregate)**						
Mean	88.441 (28.747)	94.982 (27.889)	−2.115	0.054	−0.26	**0.613**
SD	17.286 (8.864)	17.718 (7.888)	−0.422	0.75	−0.052	**0.519**
**Lower extremities (aggregate)**						
Mean	53.464 (18.582)	59.890 (26.054)	**−3.051**	**0.006**	−0.376	**0.760**
SD	6.547 (5.011)	6.473 (5.404)	0.112	0.911	0.014	**0.483**
**Head (aggregate)**						
Mean	30.697 (11.609)	33.138 (10.933)	**−3.046**	**0.006**	−0.375	**0.837**
SD	6.164 (3.172)	7.127 (3.326)	**−3.332**	**0.005**	−0.41	**0.744**

Bold values represent significant effects.

Abbreviations: GTK, get to know; MAN, managerial conflict.

#### 3.1.4 Orientation Matching: Alignment of Head Postures

Table 4 shows the results for the comparison of the horizontal and vertical head orientations. Alignment of the head orientations was quantified via RWTLCC, again issuing two parameters: peak correlation and peak correlation lag. In contrast to the RWTLCC applied to the dynamic data, these parameters do not refer to the alignment of activity levels but to the similarity of each partner's head orientation, operationalized as deviation from the direct line of view (see Supporting Information). Only one significant difference was found for the peak correlation of the horizontal head orientation, which—consistently with the analysis of the horizontal head motion—revealed larger peak correlation levels for the conflict interactions. No significant correlations across conditions were found for any of the parameters.

**TABLE 4:** Paired *t*‐test comparisons (FDR corrected) of horizontal and vertical head orientation relative to the partner (rolling‐window time‐lagged cross correlations, or RWTLCCs).

	Means (SD)		Paired *t*‐test			
Variables	Initial (GTK)	Conflict (MAN)	*t*(65)	*p*	Cohen's *d*	Pearson *r*
**Head orientation (horizontal)**						
Peak correlation	0.125 (0.053)	0.151 (0.054)	**−3.157**	**0.008**	−0.389	0.177
Peak correlation lag	3.076 (1.220)	3.285 (1.256)	−1.045	0.6	−0.129	0.151
**Head orientation (vertical)**
Peak correlation	0.146 (0.056)	0.148 (0.067)	−0.132	0.895	−0.016	0.157
Peak correlation lag	2.955 (1.418)	3.106 (1.290)	−0.588	0.744	−0.072	−0.175

Bold values represent significant effects.

Abbreviations: GTK, get to know; MAN, managerial conflict.

#### 3.1.5 Influence of Dyad Gender on Synchrony/Coordination

As the current study treated the dyad as the smallest unit of analysis, we did not consider gender of the individual participants as independent variable. Instead, we categorized the dyad gender as either, male (*n* = 10), female (*n* = 27), and mixed (*n* = 29). While we formulated no explicit hypotheses about the influence of gender on synchrony/coordination, respectively, it is interaction with the situation, gender in general is one of the most noted variables influencing nonverbal behavior [62]. Therefore, we conducted an exploratory analysis to identify potential dyad gender effects on our behavioral variables. Specifically, we conducted repeated measurement ANOVAs with dyad gender (male, female, mixed) as between subject factor and interaction task (GTK interaction vs. MAN interaction) as within‐subject factor. We found neither a main effect of dyad gender nor an interaction effect with the task. The complete set of FDR‐corrected results can be found in Supporting Information 5.

### 3.2 Results for the Evaluative Measures

#### 3.2.1 Interactant Impression Ratings

**FIGURE 5:**
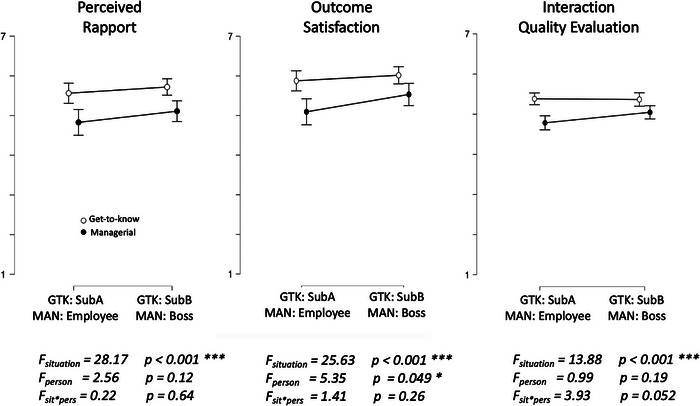
Interactant's interaction evaluation by situation and role. Panels show the interactants' subjective perceptions of rapport (left), outcome satisfaction (middle), and interaction quality (right) vary as a function of the role in the conversation across the get‐to‐know (GTK) versus managerial conflict (MAN) interactions. Note that, during the GTK task, the subjects were labeled as SubA and SubB. SubA would later become the boss and SubB the employee in managerial conflict interaction.

To examine participants’ subjective perceptions of rapport, satisfaction with the interaction outcome, and general interaction evaluation, we first compared these measures between interactants and tasks. As shown in Figure 5, there is a marked effect of the situation, with ratings of rapport, satisfaction, and interaction quality being generally higher for the GTK compared to the managerial task.

In addition to the standard Person × Situation analysis via a 2 × 2 repeated measures analysis (see Figure 5), we also examined interactants’ perceptions in specific situations via planned paired‐sample *t*‐tests. As expected, we found that during the initial GTK interaction, there were no differences in perceived rapport between the two interactants (*t* = 1.096; *p* = 0.277), nor for the satisfaction with the interaction (*t* = 0.806; *p* = 0.423) or the overall interaction quality evaluation (*t* = −0.155; *p* = 0.878). These perceptions shifted after the managerial interaction. Specifically, after the managerial interaction, participants who were in the boss role, perceived the interaction to have higher quality compared to the employee's perception (*t* = 2.113, *p* = 0.038) and showed a tendency to be more satisfied with the outcome (*t* = 1.985, *p* = 0.051). Although perceptions of rapport were not significantly different (*t* = 1.335, *p* = 0.187), the means pointed in the same direction. Furthermore, zooming in on the shifts in perceptions, as individuals transitioned from the GTK task toward the managerial task, we find that across the board, that all perceptions shifted significantly in the negative direction from the GTK to the MAN task (all *t*’s > 2.9, *p* < 0.005).

#### 3.2.2 Observer Impression Ratings

Due to technical issues with .mp4 stimuli two dyadic conflict interactions were lacking observer rating data for one of the interaction partners. These were recoded by a different coder group. The calculation of the intercoder agreement is therefore based on the 130 complete datasets only. Pearson correlations were conducted for 130 dyadic interactions and six coders using the mean rapport ratings of the 1‐min sequences.^4^ Following the rationale of intersubject correlation (ISC, [63]) the values of each coder were correlated with the means across the other five coders resulting in six correlations per stimulus. The coefficients were then Fisher z‐transformed, averaged and retransformed. Analysis resulted in a significant correlation across all stimuli (*r* = 0.402 *p* < 0.001).

**FIGURE 6:**
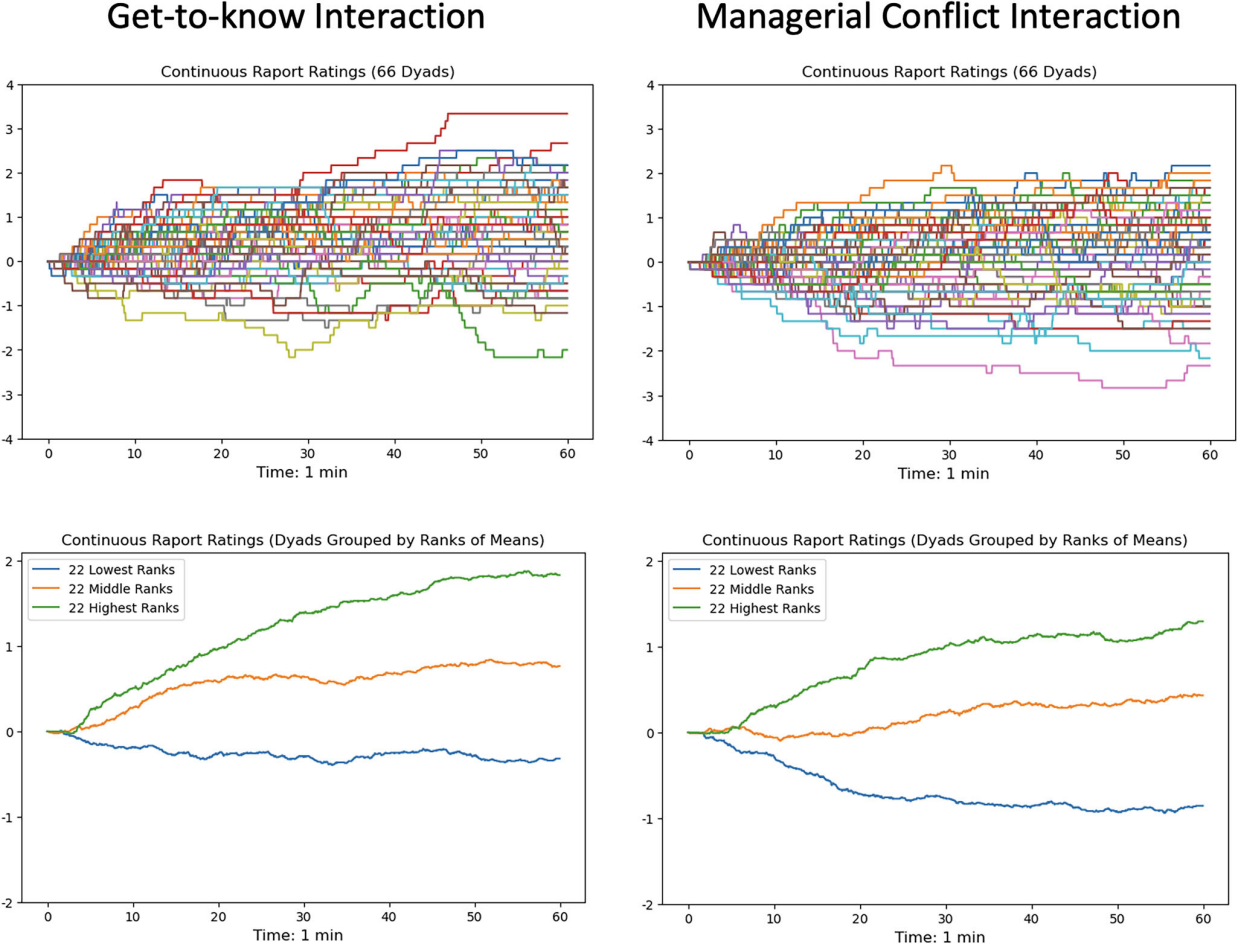
Time course of averaged continuous rapport ratings (six observers) for both interaction situations. Upper row, individual dyads; lower row, averaged for groups (22 dyads each) with the highest, middle, and lowest mean ratings.

Figure 6 displays the rating timelines of the continuous rapport rating for both conditions averaged across 6 observes. The upper row depicts the ratings for the individual dyads. The lower row depicts the rating timelines averaged for the 22 dyads with the lowest, the middle and the highest ranking in perceived rapport for each condition. As earlier reported by Bente et al. [54] the continuous ratings mostly show an asymptotic trend with few oscillations during the observation period. This is particularly evident in the rank group averages (Figure 6: lower row). The rank averages also reveal that the perceived rapport levels in all rank groups of the GTK interaction are higher than those in MAN interaction. Even the group perceived as lowest in rapport reaches an average value around the neutral scale midpoint (0) in the GTK interactions, compared to a value close to −1 in the conflict interactions while the group of the highest ranked dyads in the GTK interaction reaches values close to +2 as compared to +1 in the conflict interaction. This observation was confirmed via *t*‐test comparisons of the rating means revealing significantly lower levels of perceived rapport in the MAN (mean = 5.107, SD = 0.681) as compared to the getting‐acquainted interaction (mean = 5.498, SD = 0.658; *t*(65) = 4.565, *p* < 0.001, *d* = 0.562).

### 3.3 Correlations Between Behavioral Measures

**FIGURE 7:**
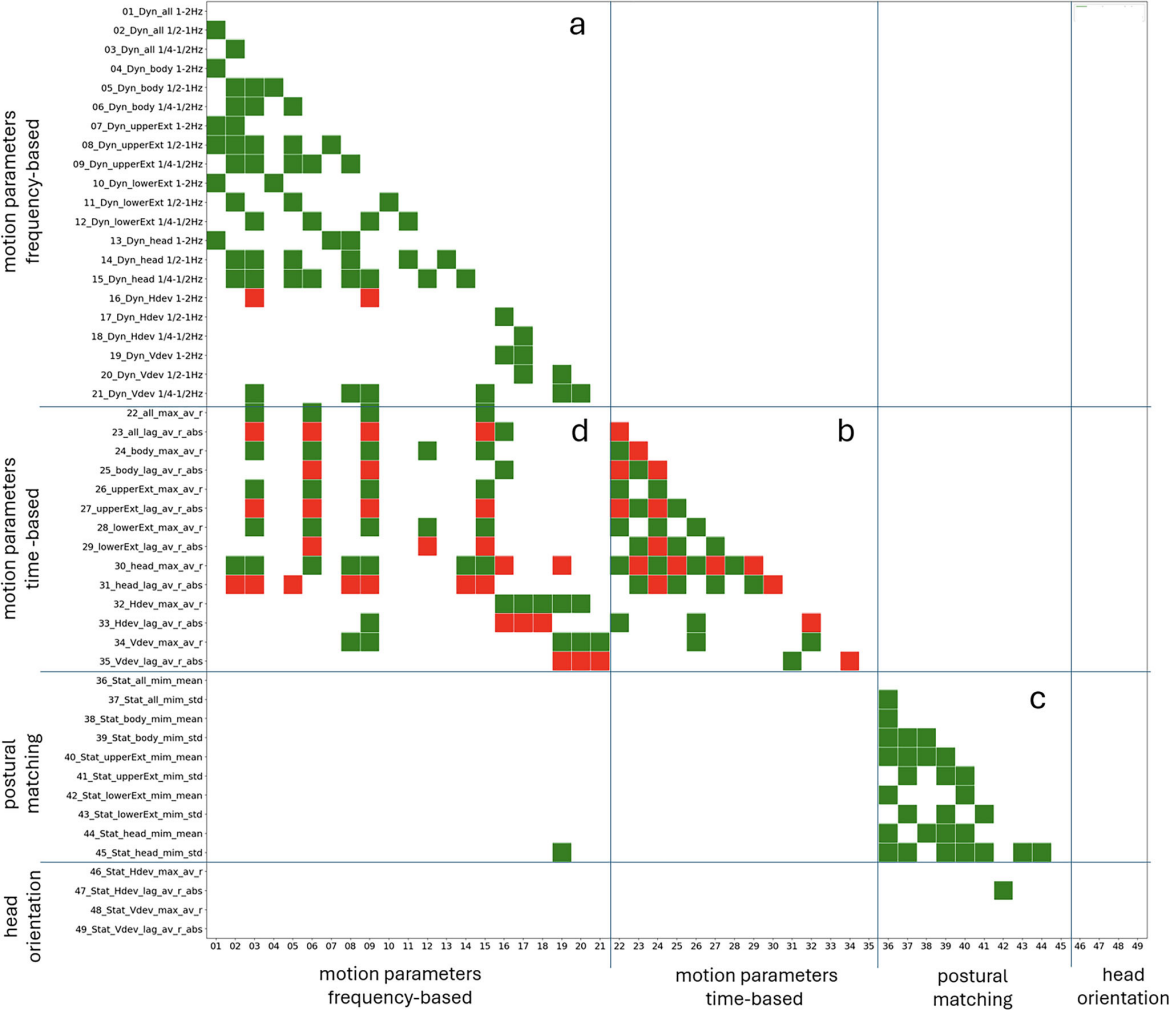
Significant cross correlation between the behavioral variables (FDR corrected, *p* < 0.05). The letters a, b, and c indicate the correlation between the parameters within one analysis procedure (frequency‐based, time‐based, and posture‐based, respectively). The letter d indicates the correlations between frequency‐ and time‐based parameters both based on dynamic motion data.

Figure 7 visualizes the intercorrelation between the various behavioral measures. Significant positive correlations (FDR corrected) are shown as green squares, negative correlation accordingly in red. It is not the room here to analyze these correlations in detail. Numeric data with the correlations are provided in Supporting Information 5. It is important to note though that frequency‐ and time‐based parameters show significant correlations within and across both methods. The postural matching parameters only correlate within this parameter set. Head orientation parameters, with one exception, neither correlate with each other nor with any of the other parameter sets.

### 3.4 Correlations Between Evaluative Measures

**FIGURE 8:**
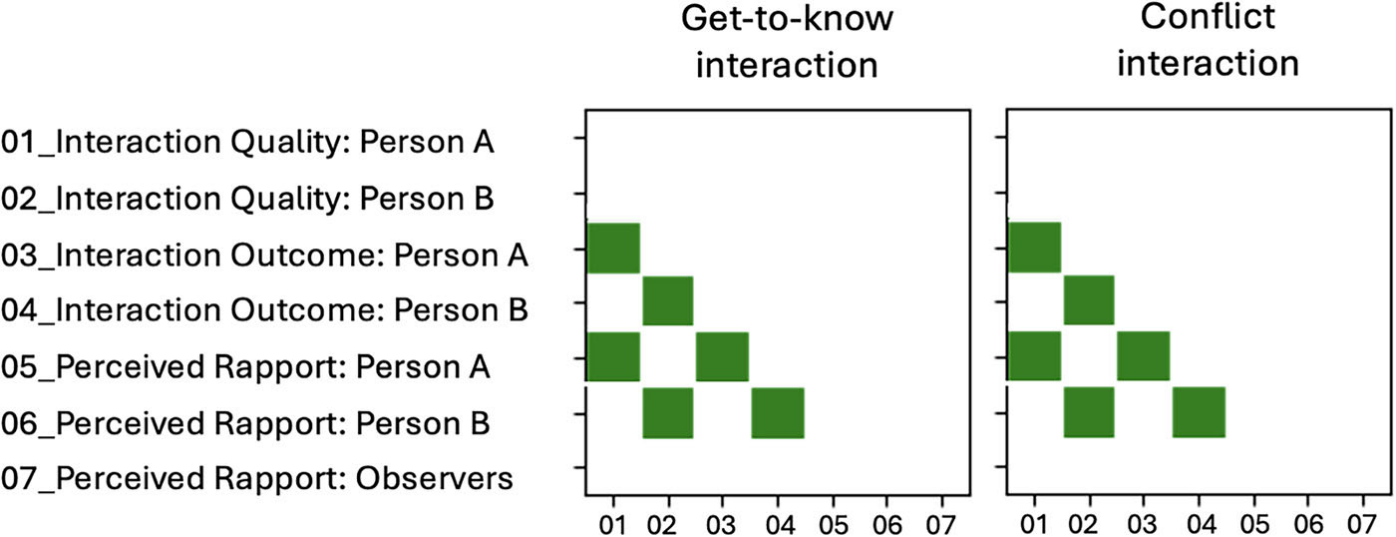
Significant cross correlation between the evaluative measures for both interaction tasks (FDR corrected, *p* < 0.05).

Figure 8 visualizes the correlations between the evaluative ratings of both interaction partners and the observers (numerical data can be found in the Supporting Information). Only significant positive correlations (FDR corrected) are displayed. As shown, no significant correlations emerged between the evaluations of the two interaction partners for any of the three rating dimensions. Likewise, none of the interactants’ ratings correlated with observers’ impressions of rapport. Positive correlations were found exclusively within each interactant across the three dimensions. The identical correlation patterns observed in both the GTK and conflict interactions suggest that each interactant's evaluation remained consistent across situations.

### 3.5 Correlations Between Behavioral and Evaluative Measures

Correlations between the behavioral and evaluative measures are detailed in the Supporting Information. Between all seven evaluative measures × 49 behavioral parameters no significant correlations were found for the GTK interaction and only two significant positive correlations were found for the conflict interaction regarding interaction quality and rapport as perceived by interactant A (boss) and the peak correlation lag of the dyads’ lower extremity movements. Despite passing the significance level after FDR correction these correlations are negligible and in fact difficult to interpret.

## 4 Discussion

This study investigated how nonverbal coordination dynamics change across two contrasting social contexts: casual initial conversations and hierarchical conflict interactions. Using high‐resolution full‐body motion capture, we examined interpersonal alignment across multiple nonverbal subsystems and coordination domains—temporal, frequency‐based, and spatial. The results paint a differentiated picture of nonverbal coordination as a multifaceted phenomenon that is both context‐sensitive and subsystem‐specific. In particular, our results reveal that different facets of nonverbal coordination respond in distinct ways to situational change, and that coordination patterns are not uniformly disrupted or enhanced under conflict but rather show subsystem‐specific modulations.

### 4.1 Summary of Main Findings

In the time domain, alignment of movement activity—as measured through RWTLCCs—was significantly reduced in the conflict interactions, particularly for trunk and head movements. Additionally, peak correlation lags increased in several subsystems, suggesting larger delays in the mutual alignment of movement activity levels. These findings are consistent with the notion that conflict situations, compared to rapport‐building contexts, may challenge spontaneous alignment and induce coordination breakdowns in some channels.

In the frequency domain, a different picture emerged. Notably, higher coherence was observed during conflict, specifically in the 1–2 Hz frequency band for vertical and horizontal head movements. The increased rhythmic entrainment in both head motion dimensions could signify convergence in the back‐channel activities (nodding and shaking) of the interaction partners or enhanced coregulation of mutual attention (head orientation toward or away from the partner) which is closely related to social gaze (see [64]). Both subtle mechanisms of interpersonal attunement may carry heightened interpersonal significance in higher stake social interactions likely being more salient and socially monitored than static postural cues, and reflect the increased pressure to manage engagement, social distance, and arousal during conflict.

In the spatial domain, postural similarity decreased significantly across nearly all body regions during the conflict task. At the same time, postural matching remained highly correlated across situations, suggesting dyad‐specific baselines in morphological coordination that persist despite contextual change. This supports the idea that postural similarity may reflect relatively stable interpersonal tendencies, potentially rooted in dyadic compatibility or mutual disposition, which become modulated—but not erased—by situational demands.

Interestingly, head orientation alignment, which was measured independently from dynamic movement, showed only marginal modulation across conditions. This may indicate that while dynamic head movements (frequency domain) become more tightly coupled during conflict, static alignment in head orientation toward the partner does not necessarily follow the same pattern, pointing to a decoupling of spatial and dynamic features in this nonverbal subsystem.

Interactants’ self‐reports indicated clear effects of the social situation, with lower ratings of rapport, satisfaction, and interaction quality during the conflict interactions. However, while each interactant's evaluations were internally consistent across rating dimensions and across conditions—suggesting stable individual judgment tendencies—there were no significant correlations between the evaluations of both interaction partners. This indicates that dyadic partners can interpret the same interaction quite differently, without necessarily developing a shared sense of rapport. This divergence was particularly pronounced in the conflict condition, where participants assigned to the boss role rated the interaction more positively than those in the employee role, especially in terms of perceived interaction quality. These role‐related asymmetries highlight how structural inequalities within an interaction may skew subjective evaluations of relational quality.

Observers’ rapport ratings also showed significant decreases from the initial conversation to the conflict interaction. Importantly, observer ratings—based solely on motion‐captured character animations without access to facial, vocal, or verbal cues—exhibited considerable consistency, with a mean inter‐rater correlation of *r* = 0.40 (*p* < 0.001). This suggests that interpersonal impressions can be reliably formed based on movement patterns alone. However, despite this consistency, observer ratings did not correlate significantly with interactants' self‐reports, pointing to a dissociation between external and internal views of the same interaction. A likely explanation lies in the difference in perceptual access: while interactants experienced the full spectrum of behavioral and verbal cues (including facial expressions, tone of voice, and spoken content), observers saw stripped‐down motion representations that abstracted away from such information.

Correlations between behavioral and evaluative data were largely absent. Only two weak associations emerged in the conflict condition after statistical correction, and these were small in magnitude and difficult to interpret theoretically. These findings suggest that spontaneous nonverbal coordination, as measured through motion capture, may not be consciously accessible or directly translated into global interaction judgments. This could speak for an approach to nonverbal coordination as an epiphenomenon with no functional role in interaction. However, it is also conceivable that the other channels available to the interactor, that is, physical appearance, voice, face, gaze, and verbal content, explain larger parts of the interaction insiders’ judgment variance.

Particularly striking though, is the lack of alignment between observer ratings and the behavioral parameters—despite both being derived from the same underlying motion data. While some socially meaningful information is clearly embedded in these motion patterns (as evidenced by inter‐rater consistency), the analytical features extracted (e.g., synchrony, postural matching) may not fully capture the cues used in social evaluation. Future work should refine these behavioral measures to better align with the perceptual salience of movement features as interpreted by both interactants and observers.

In summary, the correlational findings of this study challenge previous conclusions regarding the alignment of self‐ and other ratings of rapport and the proposed role of nonverbal coordination dynamics in shaping these impressions. For instance, Bernieri et al. [65], using a lens model framework, reported strong agreement between self‐ and observer ratings of rapport, and identified nonverbal synchrony as one significant predictors of these judgments. However, two key differences distinguish their study from the present work: (a) observers in their study had access to video and audio recordings, thus receiving multimodal input, and (b) behavioral indicators were based on human coding rather than continuous, sensor‐based measurement.

On the one hand, judges’ access to full audiovisual input may explain the greater convergence in the rapport ratings of participants and observers in their study. On the other hand, as Cappella [66] already noted, coding the nonverbal coordination may be subject to confounds: “Judgments of coordination, whether by participants or observers, could be confounded with judgments of positivity if judges' implicit theories of social interaction are that positive interactions are ones in which the people are in sync. If this is the case, then the judges would be assessing positivity and not synchrony, and the correlation to rapport would be an artifact” (p. 303).

Taken together, these findings directly speak to the four guiding questions of this study. First, we found clear evidence that interpersonal coordination dynamics change when dyads move from a casual conversation to a hierarchical conflict, with subsystem‐specific modulations observed across all coordination domains. Second, the correlation of postural similarity across conditions suggests that initial dyadic coordination may indeed covary with coordination patterns during conflict, reflecting dyad‐specific baseline tendencies. Third, however, behavioral coordination showed only spurious correlations with subjective evaluations of perceived rapport, satisfaction, or interaction quality. Finally, our differentiated results demonstrate that various coordination measures—spanning time, frequency, and spatial domains—respond in distinct ways to social context and show little convergence in their relation to evaluative judgments.

### 4.2 Theoretical Implications

Perhaps the most important conceptual takeaway from this study is that it challenges the notion of nonverbal synchrony as a unitary construct. Instead, our findings underscore the need to differentiate between distinct movement subsystems and coordination facets—each defined by their rhythmic, temporal, or spatial characteristics [10, 15, 35, 42]. Coordination is not simply more or less present—it manifests in divergent forms, each potentially serving different communicative or relational functions. For instance, under conflict conditions, we observed a decrease in peak correlation for trunk motion but an increase for head motion. This divergence suggests that different nonverbal channels may serve distinct regulatory functions in social interaction, especially in stressful or asymmetric contexts. Whereas body posture—particularly trunk alignment—may reflect more stable, role‐related positioning, head movements may be more dynamically responsive, supporting moment‐to‐moment regulation of attention and engagement. This interpretation aligns with previous findings from psychotherapy research showing functional differences between head and body coordination [67].

Overall, this study proposes a refined conceptual framework for describing interpersonal alignment in nonverbal behavior. We suggest using interpersonal coordination as an overarching construct that encompasses distinct mechanisms, while reserving synchrony for the entrainment of movement rhythms—typically captured by frequency‐based measures and most relevant when rhythmic properties are present or expected in nonverbal communication. Within this broader coordination framework, we propose to differentiate three key facets: (1) rhythmic synchrony in movement patterns (e.g., head nods or beat gestures), (2) temporal alignment of activity levels over time, and (3) spatial matching, further divided into postural similarity (based on translational data) and orientation alignment (based on rotational data).

Our data suggest that each facet may serve different functions in social interaction. Postural matching may reflect a more enduring and situationally grounded form of coordination, capturing trait‐like preferences or sociostructural relationships (e.g., equality or hierarchy). This is supported by our finding that postural similarity—particularly in the trunk, the most stable body segment—decreased under hierarchical conflict while retaining strong correlations across dyads, suggesting both task sensitivity and dyad‐specific baselines. In contrast, frequency‐based synchrony in faster movement oscillations, such as horizontal head motion, may track more immediate and relationally relevant dynamics, particularly those associated with mutual attention and engagement. These faster oscillations likely unfold in concert with the turn‐by‐turn structure of conversation and may become more salient—consciously or unconsciously—in high stakes or emotionally loaded interactions such as conflict, where the cost of misalignment is higher.

Temporal alignment of movement activity—assessed through time‐lagged cross correlations—on the other hand more likely may reflect an emergent property of a harmonic dyadic system rather than a functional mechanism in itself. Its relatively weaker situational sensitivity and sparse links to evaluative outcomes in our data raise questions about whether such metrics capture actively regulated behavior or merely mirror more fundamental aspects of interpersonal alignment.

Together, these distinctions underscore the value of abandoning the umbrella use of synchrony and instead adopting *coordination* as the broader construct, with specific operational definitions tied to both analytic strategies and hypothesized social functions.

### 4.3 Limitations and Future Directions

While this study leveraged high‐resolution motion capture and a robust within‐dyad design, several limitations warrant consideration. First, the conflict interaction was scripted and role‐assigned, which—although ecologically valid in terms of task structure—may limit generalizability to naturally emerging disagreements. Relatedly, the getting‐to‐know‐each‐other task always preceded the managerial task, raising the possibility of order effects. Second, while our analytical framework focused on nonverbal behavior, it did not yet incorporate the rich verbal and physiological data that were also collected as part of the broader dataset. These additional modalities will be revisited in the next phase of our research to address open questions about how verbal exchange and autonomic synchrony interact with nonverbal coordination processes. Future analyses may also focus on longer interaction sequences to capture evolving dynamics over time and link fluctuations in behavioral alignment to shifts in communication content and relational framing.

Another important consideration concerns the data acquisition method: while motion capture offers high spatial and temporal precision, it may be somewhat obtrusive and less scalable. Emerging video‐based motion analysis tools, such as OpenPose [44, 68] and other markerless pose estimation techniques, offer promising alternatives for future research. These tools not only reduce participant burden but also open the door to studying naturally occurring interactions captured in large video databases—such as televised political debates, job interviews, or clinical encounters—thereby enhancing ecological validity and generalizability.

While many methods exist to quantify nonverbal coordination or temporal interdependencies, a major challenge lies in the multidimensional nature of the raw behavioral data. Our findings highlight a key disconnect: despite consistent observer judgments based on motion alone, these impressions did not correlate with specific coordination metrics extracted from the same data. This suggests that humans may integrate information across multiple behavioral channels—or flexibly shift between them—depending on where socially meaningful dynamics are most salient. Thus, psychological interpretations of coordination may not map cleanly onto singular or isolated behavioral indicators. Future research should consider machine learning and multivariate modeling approaches that can better capture and disentangle the complex, layered interdependencies underlying interpersonal coordination and its perceived meaning.

### 4.4 Conclusion

In sum, this study demonstrates that interpersonal coordination is a multifaceted and dynamic phenomenon. Different interaction contexts and social demands evoke differential patterns of alignment and misalignment across movement dimensions and coordination domains. Rather than viewing synchrony as a singular process, our findings support a layered conceptualization of coordination, sensitive to bodily subsystems, analytic perspective, and situational constraints. This nuanced view provides a promising foundation for future research aimed at “reading the social clock”—deciphering how interpersonal coordination dynamics unfold in real time and across changing social terrains.

## Author Contributions

**Gary Bente**: Conceptualization, methodology, software, formal analysis, investigation, resources, data curation, writing—original draft, writing—review and editing, visualization, supervision, project administration, funding acquisition, study design, data collection, statistical analysis, data interpretation, and contributed reagents/materials/analysis tools. **Ralf Schmälzle**: Conceptualization, methodology, software, formal analysis, investigation, resources, data curation, writing—original draft, writing—review and editing, visualization, supervision, project administration, funding acquisition, study design, data collection, statistical analysis, data interpretation, and contributed reagents/materials/analysis tools. **Nolan Jahn**: Project administration, data collection, and writing—review and editing. **Mark Reimers**: Software, formal analysis, writing—review and editing, visualization, funding acquisition, statistical analysis, and data interpretation.

## Supporting information



Supporting Information: nyas70118‐sup‐0001‐SuppMat.docx

## Data Availability

The data that support the findings of this study are available upon request from the corresponding author. The data are not publicly available due to privacy or ethical restrictions.

## Footnotes

1 Being aware of the multiple definitions, and the broad variety of metrics applied, we for now use *interpersonal synchrony* and *coordination* synonymously as umbrella terms encompassing “all instances when the states of two or more interacting people are related over time” ([10], p. 4). We concur with the authors that such a *“maximally inclusive definition”* (ibid) necessitates the specification of the forms or instances of synchrony under investigation, which is a goal of this study.

2 Verbal, physiological, and eye tracking data are reported elsewhere.

3 The complete set of survey instruments and ratings scales can be found in Supporting Information 3.

4 Due to technical issues with .mp4 stimuli two dyadic conflict interactions were lacking observer rating data for one of the interaction partners. These were recoded by a different coder group. The calculation of the intercoder agreement is therefore based on the 130 complete datasets only.
