# Supplementary material for: Reading the Social Clock: Analyzing Nonverbal Coordination Dynamics in Casual Chat and Conflict
Source: Ann N Y Acad Sci. 2025 Oct 26;1554(1):121–39. doi: 10.1111/nyas.70118 (PMC12728332; doi:10.1111/nyas.70118)
Supplement: Supplementary file 1 — Supporting Information: nyas70118‐sup‐0001‐SuppMat.docx [file NYAS-1554-121-s001.docx]

**Supplementary Materials**

**Supplement 1 – Measurement of Head Orientation**

The current study aimed to include head orientation relative to the interaction partner to reveal information about the coordination dynamics in mutual attention and backchannel activity (head nods, shakes). As Euler angles representing the rotation of the neck joint within the ‘.fbx’ animation files are not a straightforward reflection of the spatial orientation of the head, we developed a custom method to extract this information by applying a ray tracing algorithm to the animation protocols. The algorithm is implemented in the python-based VR-platform Vizard 7.0. The program uses the translation and rotation information from the exported skeleton data sets to play back the dyadic head motions in fame-by-fame mode. It also adds two virtual rays on the forehead center of each of the heads pointing towards the respective counterpart. Deviations of the ray’s endpoint of one person to the partner’s ray origin were then calculated, adjusting the ray length continuously to match the distance of the two origins. Horizontal and vertical deviations (cm) and deviation angles (degrees) were stored as continuous orientation parameters. Figure S1 illustrates the rationale of the program.

| 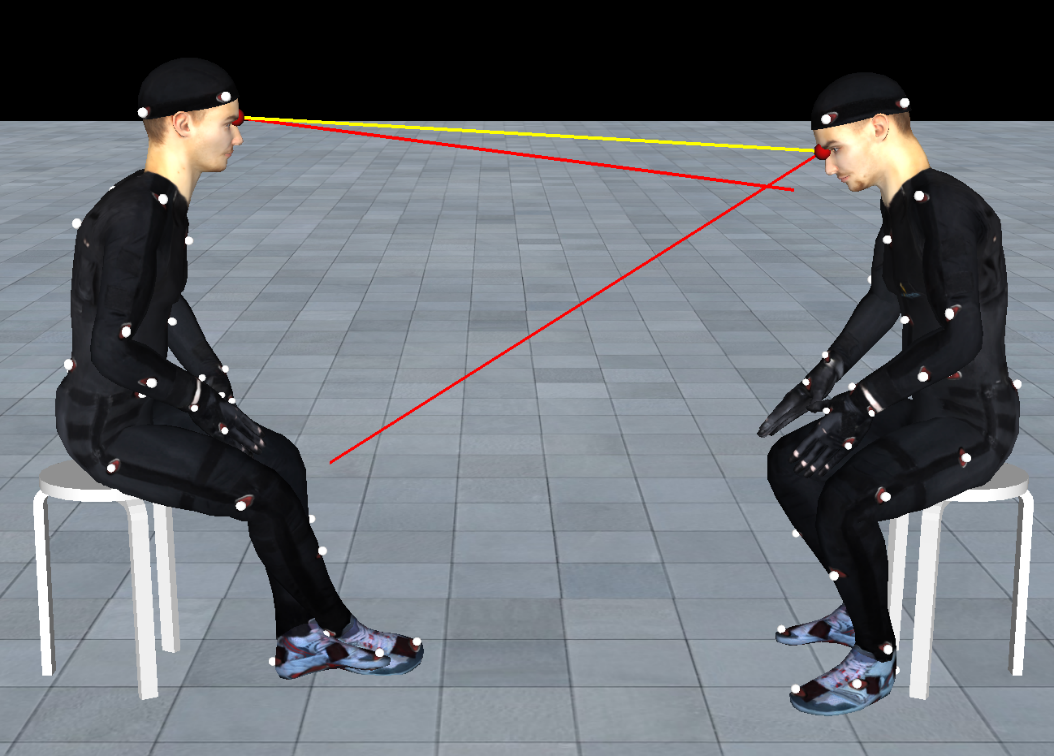**Head Orienation Tracking (Vizard 3.0 Python)** |
| --- |

***Figure S1: Screenshot illustrating the rationale of the ray tracing algorithm applied to the extraction of partner-oriented head orientations.*** *The figure shows the full body version of the program. For reasons of processing speed, only head animations were used for the final data export for all 132 dyads.*

**Supplement 2 – Measurement of Postural Similarity**

Following recent practice (Bente et al. 2020; Novotny & Bente, 2022), *Intersubject Postural Similarity* (IPS) was calculated for each time point as aggregate difference between both interactants’ standardized skeletal positions. For this purpose, the individual translation matrices were resampled to 10Hz. The 3D-coordinates of all joints at all time points were then standardized by subtracting the root joint coordinate (hips) creating a common spatial origin for both interactants (see Novotny & Bente, 2022). Further the x and z coordinates for actor B were inverted so both actors faced the same direction and right and left sides of the bodies were correctly aligned. For each data point we then calculated the Euclidian distance between both interactants’ corresponding joints. All joint distances were then added up for each data point resulting in a one-dimensional *postural similarity time series.* Figure S2 illustrates the principle of the IPS routine.

| **Postural Similarity (Python: Spyder 5.4)** | |
| --- | --- |
| 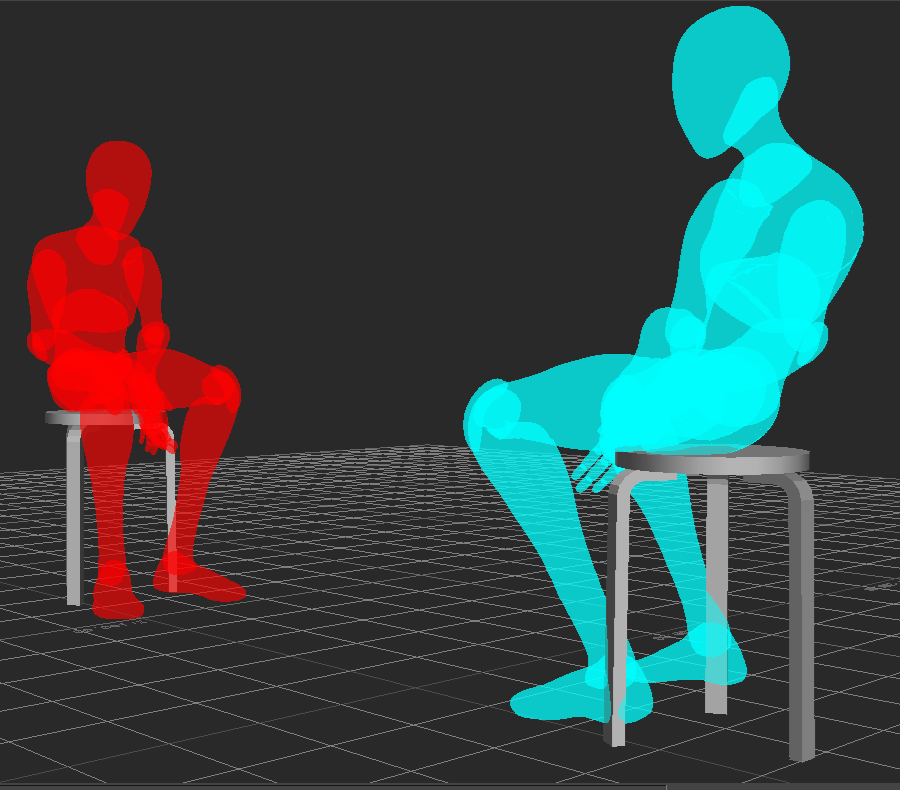 | 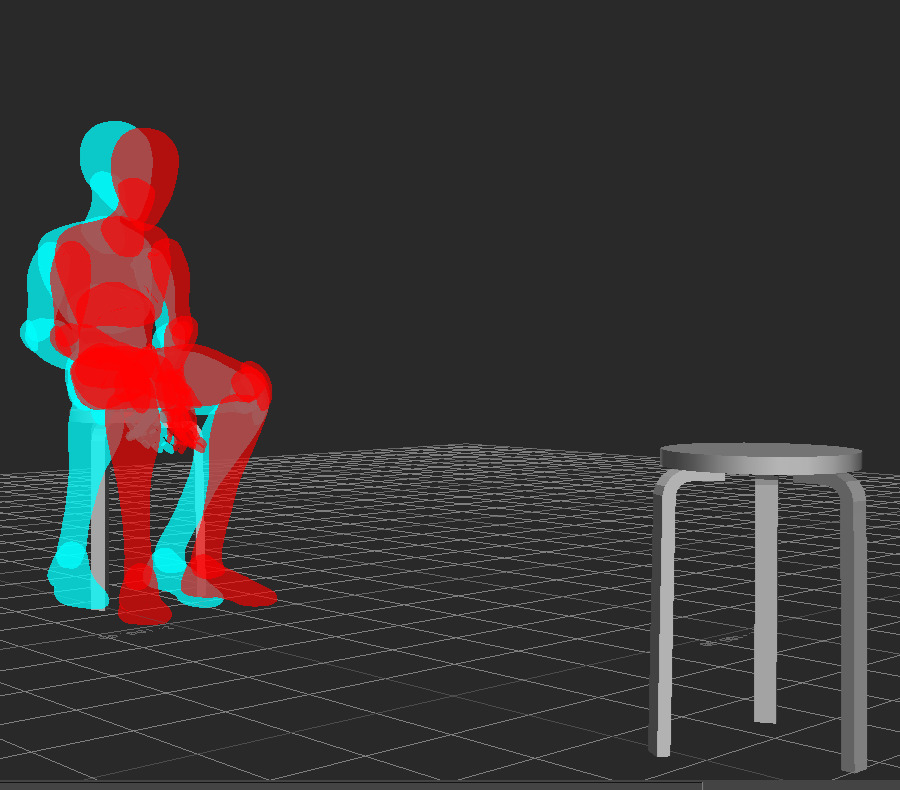 |

***Figure S2: Screen shot illustrating the rationale of the Intersubject Postural Similarity (IPS) algorithm.*** *Left: Original position in which interactants sit facing each other. Right: By moving and rotating interactant B onto the origin position of interactant A, it is possible to assess the difference in postural similarity.*

**Supplement 3 – Overview over the Survey Measures and Rating Scales**

Table S3 lists the self-report and rating instruments administered before, during and after the interaction sessions. The data will part of future research based of this database. It will not be used in the study presented below and not further detailed in the following. referred to is available on qualified request.

***Table S3. Self-report and ratings instruments administered before, during and after the interaction sessions.***

| **Time Point** | **Instrument** | **Description** | **Source** |
| --- | --- | --- | --- |
| Pre-Study | Age | Participant's age | - |
|  | Sex/Gender | Sex/Gender (self-identified) | - |
|  | Ethnicity | Ethnicity (self-identified) | - |
|  | Education | Education (self-identified) | - |
|  | Short Personality Scale: Big-Five Inventory Short form | Short version of the Big-Five inventory (10 items) | Rammstedt, B., & John, O. P. (2007). Measuring personality in one minute or less: A 10-item short version of the Big Five Inventory in English and German. Journal of research in Personality, 41(1), 203-212. |
|  | Panas-SF | Positive and negative affect scale (Short form, 10 items) | Watson, D., Clark, L. A., & Tellegen, A. (1988). Development and validation of brief measures of positive and negative affect: the PANAS scales. Journal of personality and social psychology, 54(6), 1063. |
|  | Self-Construal | Independent and Interdependent Self-Construal (30 items) | Singelis, T. M. (1994). The measurement of independent and interdependent self-construals. Personality and Social Psychology Bulletin, 20(5), 580–591. |
|  | IIP | Inventory of Interpersonal Problems (32 items) | Barkham, M., Hardy, G.E., and Startup, M. The IIP-32: A short version of the inventory of interpersonal problems |
| Post-Interaction Survey  (applied after both interaction) | Interaction Evaluation Survey: Self perception  Interaction perception  Partner perception | A mix of terms from Bernieri, Davis, Rosenthal, & Knee, 1994, complemented by our own items. 11 items for self-perception, 18 items for interaction perception, and 3 for partner perceptions) | Bernieri, F. J., Davis, J. M., Rosenthal, R., & Knee, C. R. (1994). Interactional synchrony and rapport: Measuring synchrony in displays devoid of sound and facial affect. Personality and social psychology bulletin, 20(3), 303-311. |
|  | Semantic Differential Self-vs.-Partner | A semantic differential-style measure of self-vs. partner evaluations for bipolar social-evaluative adjective pairs (20 items) | Bente, G., Leuschner, H., Al Issa, A., & Blascovich, J. J. (2010). The others: Universals and cultural specificities in the perception of status and dominance from nonverbal behavior. Consciousness and cognition, 19(3), 762-777. |
|  | IES:  Self perception  Interaction perception  Partner perception | Interaction Evaluation Survey: A mix of terms from Bernieri, Davis, Rosenthal, & Knee, 1994, complemented by our own items. 11 items for self-perception, 18 items for interaction perception, and 2 for partner perceptions) |  |
|  | Semantic Differential Self-vs.-Partner | A semantic differential-style measure of self-vs. partner evaluations for bipolar social-evaluative adjective pairs (20 items) | Bente, G., Leuschner, H., Al Issa, A., & Blascovich, J. J. (2010). The others: Universals and cultural specificities in the perception of status and dominance from nonverbal behavior. Consciousness and cognition, 19(3), 762-777. |
| Post-Managerial Task | Interaction Evaluation Survey  Self perception  Interaction perception  Partner perception | A mix of terms from Bernieri, Davis, Rosenthal, & Knee, 1994, complemented by our own items. 11 items for self-perception, 18 items for interaction perception, and 2 for partner perceptions) |  |
|  | Inclusion of Others and Self | A one-item pictographic rating scale in which circles that overlap to varying degree can be selected to indicate the degree of experienced self-other overlap | Aron, A., Aron, E. N., & Smollan, D. (1992). Inclusion of other in the self scale and the structure of interpersonal closeness. Journal of Personality and Social Psychology, 63(4), 596-612. |
|  | Self-Assessment Manikin | A 3-item graphical rating scales in which manikins can be selected to reflect the degree of experienced valence, arousal, and dominance | Bradley, M. M., & Lang, P. J. (1994). Measuring emotion: the self-assessment manikin and the semantic differential. Journal of behavior therapy and experimental psychiatry, 25(1), 49-59. |
|  | Panas-SF |  |  |
|  | Future interaction willingness | Willingness to meet the interaction partner again |  |
| Post Study Survey | Interaction naturalness | Degree to which the interaction felt natural |  |
|  | Comfort during study | Degree of comfort experienced during the study |  |
|  | Impairment by technology | Perceived impairment of natural movement during the study |  |
|  | Willingness to participate again | Willingness to participate again in the study |  |

**Supplement 4 – Stimulus Production for the Observer Study**

The current study uses character animations of neural, standardized avatars instead of video as stimuli for observer ratings to avoid influences of physical appearance on impression formation. Figure S4 shows a sequence of screen shots illustrating the workflow of the stimulus production. A custom python program realized as MotionBuilder 2024 plugin was developed to automatize the otherwise very labor intense production process. The software specifically automatizes the multiple steps of the character animation process that is otherwise very labor intense. Character animations for all 132 interactions thus could be performed in a loop without any manual intervention. The program first loads the ‘.fbx’ skeleton animations exported from the capture software Motive 3.0 into MotionBuilder. It then performs a characterization and a T-pose correction of the included skeleton models. It then imports both avatar models (a rigged character from Mixamo.com) and performs their characterization. The avatar models (targets) are then re-targeted to the skeletons (sources). We finally hand edited the resulting avatar animations to correct for major flaws, such as hand penetrations of thighs or body and floor contact. After completion of this process the animations were again stored as ‘.fbx’-files. These were then imported by Unreal Engine that was used to render the first minute of each interaction in high resolution into .mp4 videos. As the role of the boss in the managerial task was always taken by the person sitting on the right in the original setting, we additionally produced side-flipped animation versions of all clips to ensure that no positional effects could occur.

| *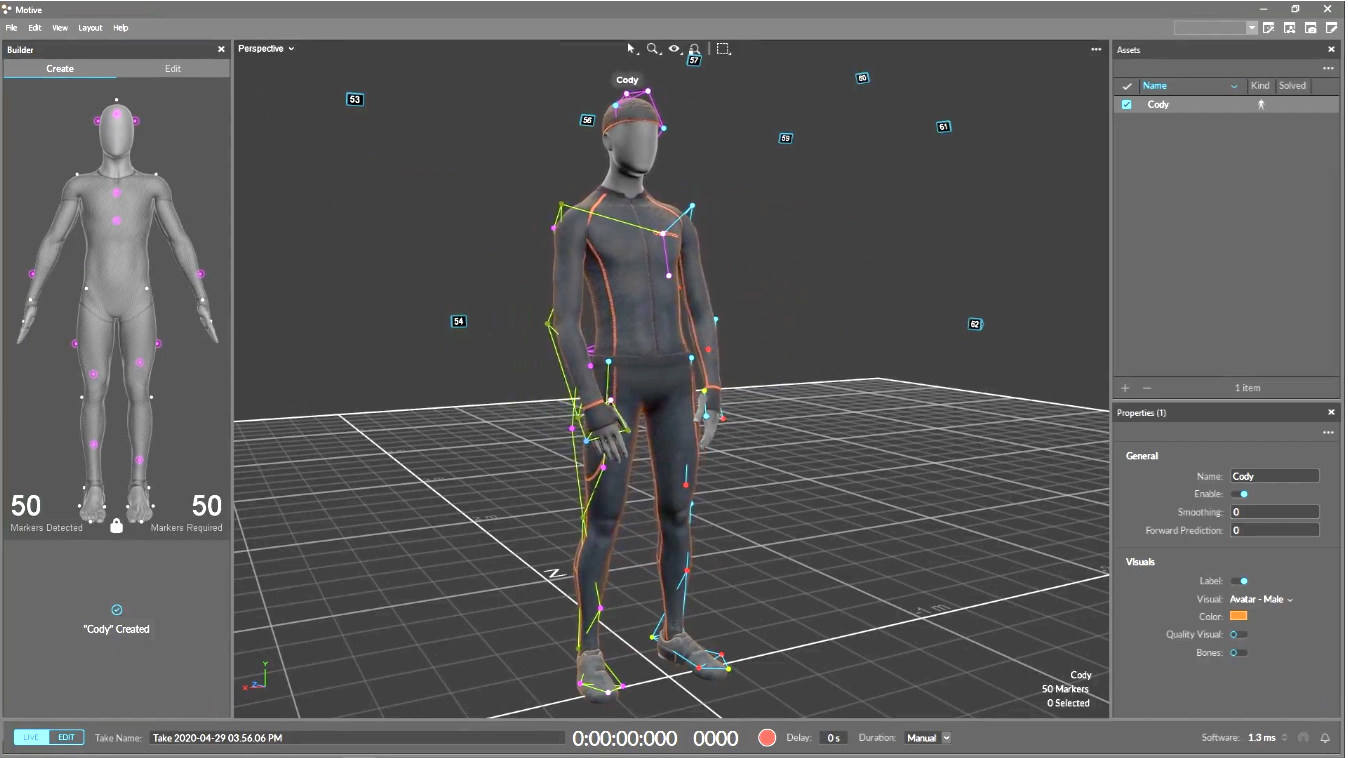***Optitrack - Motive 3.0 : Motion Capture** |
| --- |
| **Motion Builder 2024: T-Posing and Characterization 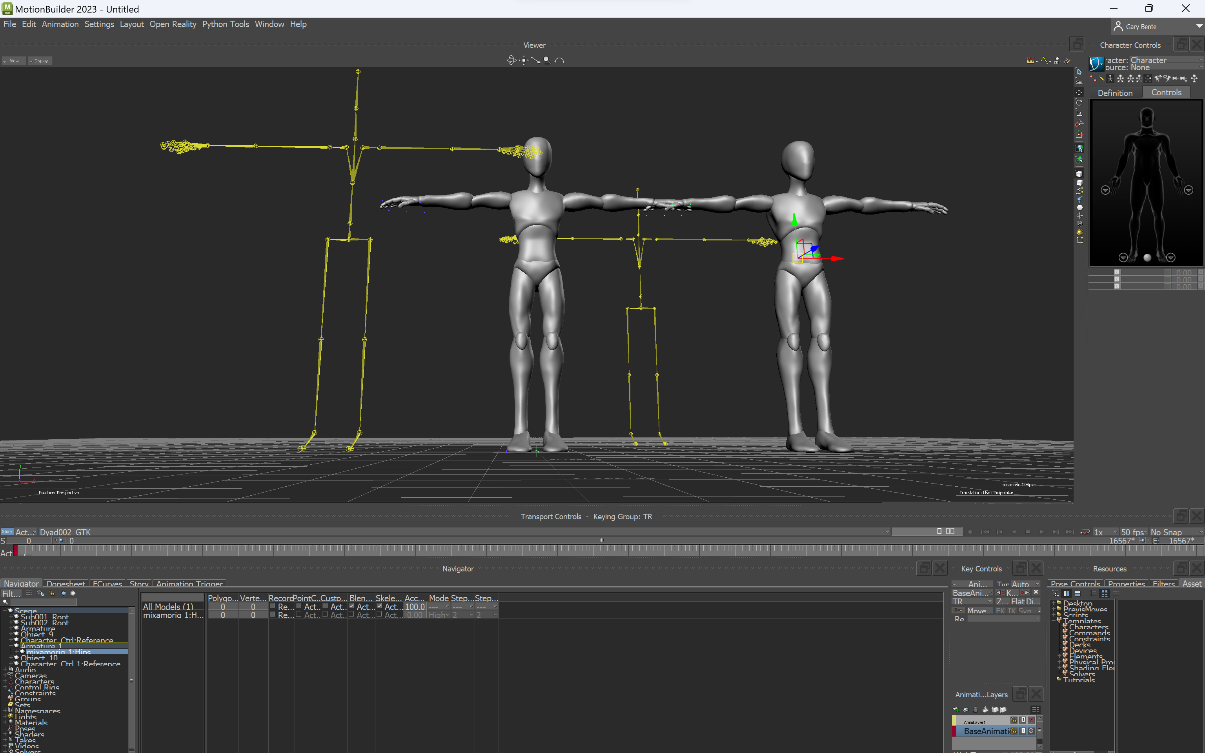** |
| **Motion Builder 2023: Final Editing and .fbx 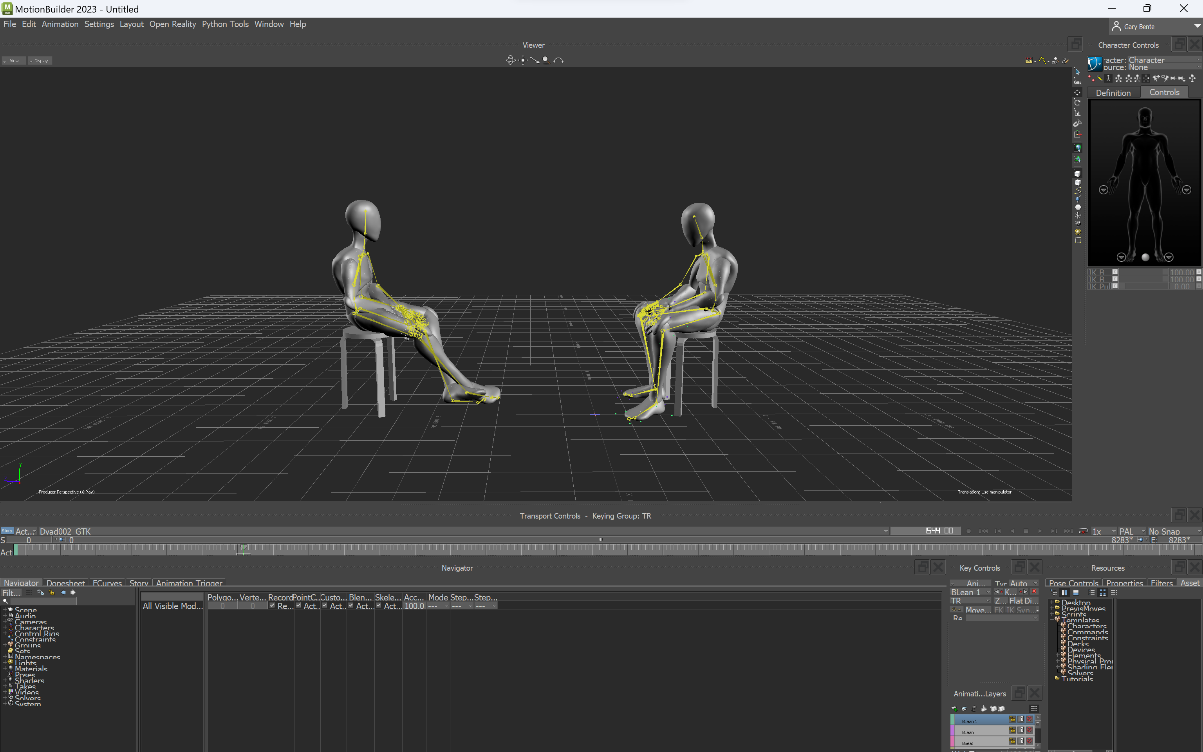Export** |
| **Ureal Engine 5.4: Rendering to .mp4 Files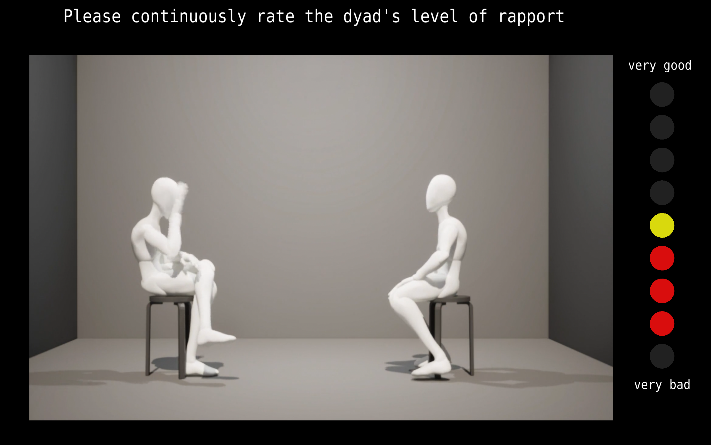 (final stimuli for onserver study)** |

***Figure S4: Production Sequence of the Character Animation Stimuli.***

*
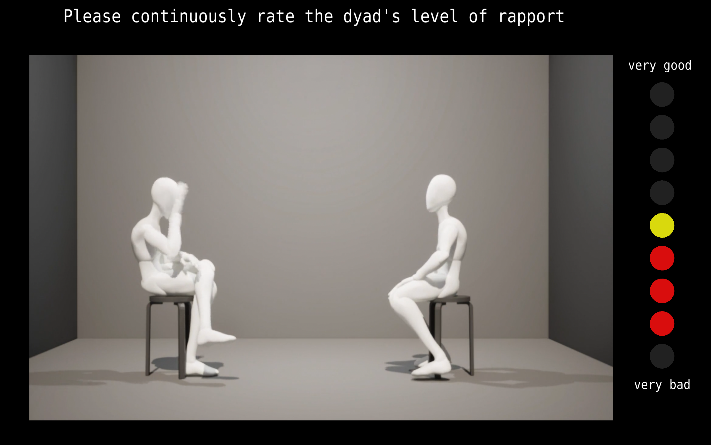
*

**Supplement 5 – ANOVA Results: Interaction Task (within) x Dyad Gender (male, female, mixed)**

| **5.1 Task x Gender: RWTLCC dynamic data** | |  |  |  |
| --- | --- | --- | --- | --- |
| Variable | Source | F | p | np2 |
| all_max_av_r | Task (Within) | 3.661 | 0.102 | 0.055 |
|  | SexDyad (Between) | 0.649 | 0.749 | 0.02 |
|  | Task * SexDyad (Interaction) | 0.16 | 0.979 | 0.005 |
| all_lag_av_r_abs | Task (Within) | 10.542 | 0.005 | 0.143 |
|  | SexDyad (Between) | 0.534 | 0.749 | 0.017 |
|  | Task * SexDyad (Interaction) | 1.128 | 0.979 | 0.035 |
| body_max_av_r | Task (Within) | 6.361 | 0.033 | 0.092 |
|  | SexDyad (Between) | 0.756 | 0.749 | 0.023 |
|  | Task * SexDyad (Interaction) | 0.155 | 0.979 | 0.005 |
| body_lag_av_r_abs | Task (Within) | 13.894 | 0.003 | 0.181 |
|  | SexDyad (Between) | 0.794 | 0.749 | 0.025 |
|  | Task * SexDyad (Interaction) | 2.161 | 0.577 | 0.064 |
| upperExt_max_av_r | Task (Within) | 2.175 | 0.195 | 0.033 |
|  | SexDyad (Between) | 1.381 | 0.738 | 0.042 |
|  | Task * SexDyad (Interaction) | 0.021 | 0.979 | 0.001 |
| upperExt_lag_av_r_abs | Task (Within) | 1.83 | 0.211 | 0.028 |
|  | SexDyad (Between) | 0.363 | 0.751 | 0.011 |
|  | Task * SexDyad (Interaction) | 0.631 | 0.979 | 0.02 |
| lowerExt_max_av_r | Task (Within) | 0.138 | 0.712 | 0.002 |
|  | SexDyad (Between) | 0.395 | 0.751 | 0.012 |
|  | Task * SexDyad (Interaction) | 0.317 | 0.979 | 0.01 |
| lowerExt_lag_av_r_abs | Task (Within) | 16.026 | 0.002 | 0.203 |
|  | SexDyad (Between) | 1.178 | 0.738 | 0.036 |
|  | Task * SexDyad (Interaction) | 2.377 | 0.577 | 0.07 |
| head_max_av_r | Task (Within) | 5.578 | 0.043 | 0.081 |
|  | SexDyad (Between) | 1.173 | 0.738 | 0.036 |
|  | Task * SexDyad (Interaction) | 0.06 | 0.979 | 0.002 |
| head_lag_av_r_abs | Task (Within) | 10.978 | 0.005 | 0.148 |
|  | SexDyad (Between) | 5.605 | 0.081 | 0.151 |
|  | Task * SexDyad (Interaction) | 0.209 | 0.979 | 0.007 |
| Hdev_max_av_r | Task (Within) | 11.795 | 0.005 | 0.158 |
|  | SexDyad (Between) | 0.081 | 0.922 | 0.003 |
|  | Task * SexDyad (Interaction) | 3.433 | 0.538 | 0.098 |
| Hdev_lag_av_r_abs | Task (Within) | 3.512 | 0.102 | 0.053 |
|  | SexDyad (Between) | 1.395 | 0.738 | 0.042 |
|  | Task * SexDyad (Interaction) | 0.186 | 0.979 | 0.006 |
| Vdev_max_av_r | Task (Within) | 2.086 | 0.195 | 0.032 |
|  | SexDyad (Between) | 1.597 | 0.738 | 0.048 |
|  | Task * SexDyad (Interaction) | 0.167 | 0.979 | 0.005 |
| Vdev_lag_av_r_abs | Task (Within) | 0.759 | 0.417 | 0.012 |
|  | SexDyad (Between) | 0.547 | 0.749 | 0.017 |
|  | Task * SexDyad (Interaction) | 0.456 | 0.979 | 0.014 |

| **5.2 Task x Gender: WCT dynamic data** | |  |  |  |
| --- | --- | --- | --- | --- |
| Variable | Source | F | p | np2 |
| all 1-2Hz | Task (Within) | 0.204 | 0.805 | 0.003 |
|  | SexDyad (Between) | 2.177 | 0.488 | 0.065 |
|  | Task * SexDyad (Interaction) | 0.514 | 0.902 | 0.016 |
| all 1/2-1Hz | Task (Within) | 0.918 | 0.553 | 0.014 |
|  | SexDyad (Between) | 0.418 | 0.924 | 0.013 |
|  | Task * SexDyad (Interaction) | 2.227 | 0.841 | 0.066 |
| all 1/4-1/2Hz | Task (Within) | 1.879 | 0.409 | 0.029 |
|  | SexDyad (Between) | 1.268 | 0.704 | 0.039 |
|  | Task * SexDyad (Interaction) | 0.735 | 0.902 | 0.023 |
| all 1/8-1/4Hz | Task (Within) | 0.099 | 0.844 | 0.002 |
|  | SexDyad (Between) | 0.523 | 0.898 | 0.016 |
|  | Task * SexDyad (Interaction) | 1.149 | 0.867 | 0.035 |
| body 1-2Hz | Task (Within) | 0.02 | 0.889 | 0 |
|  | SexDyad (Between) | 0.677 | 0.874 | 0.021 |
|  | Task * SexDyad (Interaction) | 2.442 | 0.841 | 0.072 |
| body 1/2-1Hz | Task (Within) | 3.272 | 0.29 | 0.049 |
|  | SexDyad (Between) | 0.246 | 0.955 | 0.008 |
|  | Task * SexDyad (Interaction) | 1.922 | 0.867 | 0.058 |
| body 1/4-1/2Hz | Task (Within) | 3.7 | 0.29 | 0.055 |
|  | SexDyad (Between) | 0.5 | 0.898 | 0.016 |
|  | Task * SexDyad (Interaction) | 0.702 | 0.902 | 0.022 |
| body 1/8-1/4Hz | Task (Within) | 1.209 | 0.515 | 0.019 |
|  | SexDyad (Between) | 0.727 | 0.874 | 0.023 |
|  | Task * SexDyad (Interaction) | 1.184 | 0.867 | 0.036 |
| upperExt 1-2Hz | Task (Within) | 1.642 | 0.441 | 0.025 |
|  | SexDyad (Between) | 2.623 | 0.488 | 0.077 |
|  | Task * SexDyad (Interaction) | 0.075 | 0.928 | 0.002 |
| upperExt 1/2-1Hz | Task (Within) | 0.161 | 0.805 | 0.003 |
|  | SexDyad (Between) | 2.649 | 0.488 | 0.078 |
|  | Task * SexDyad (Interaction) | 1.106 | 0.867 | 0.034 |
| upperExt 1/4-1/2Hz | Task (Within) | 0.777 | 0.562 | 0.012 |
|  | SexDyad (Between) | 2.278 | 0.488 | 0.067 |
|  | Task * SexDyad (Interaction) | 0.821 | 0.902 | 0.025 |
| upperExt 1/8-1/4Hz | Task (Within) | 0.033 | 0.887 | 0.001 |
|  | SexDyad (Between) | 0.32 | 0.955 | 0.01 |
|  | Task * SexDyad (Interaction) | 0.538 | 0.902 | 0.017 |
| lowerExt 1-2Hz | Task (Within) | 3.584 | 0.29 | 0.054 |
|  | SexDyad (Between) | 2.409 | 0.488 | 0.071 |
|  | Task * SexDyad (Interaction) | 2.816 | 0.841 | 0.082 |
| lowerExt 1/2-1Hz | Task (Within) | 0.866 | 0.553 | 0.014 |
|  | SexDyad (Between) | 0.185 | 0.955 | 0.006 |
|  | Task * SexDyad (Interaction) | 0.332 | 0.928 | 0.01 |
| lowerExt 1/4-1/2Hz | Task (Within) | 0.196 | 0.805 | 0.003 |
|  | SexDyad (Between) | 0.083 | 0.955 | 0.003 |
|  | Task * SexDyad (Interaction) | 0.089 | 0.928 | 0.003 |
| lowerExt 1/8-1/4Hz | Task (Within) | 0.48 | 0.688 | 0.008 |
|  | SexDyad (Between) | 0.082 | 0.955 | 0.003 |
|  | Task * SexDyad (Interaction) | 0.206 | 0.928 | 0.006 |
| head 1-2Hz | Task (Within) | 0.936 | 0.553 | 0.015 |
|  | SexDyad (Between) | 1.812 | 0.601 | 0.054 |
|  | Task * SexDyad (Interaction) | 0.133 | 0.928 | 0.004 |
| head 1/2-1Hz | Task (Within) | 3.808 | 0.29 | 0.057 |
|  | SexDyad (Between) | 1.165 | 0.704 | 0.036 |
|  | Task * SexDyad (Interaction) | 0.226 | 0.928 | 0.007 |
| head 1/4-1/2Hz | Task (Within) | 2.63 | 0.29 | 0.04 |
|  | SexDyad (Between) | 1.138 | 0.704 | 0.035 |
|  | Task * SexDyad (Interaction) | 1.147 | 0.867 | 0.035 |
| head 1/8-1/4Hz | Task (Within) | 2.65 | 0.29 | 0.04 |
|  | SexDyad (Between) | 0.64 | 0.874 | 0.02 |
|  | Task * SexDyad (Interaction) | 0.495 | 0.902 | 0.015 |
| Hdev 1-2Hz | Task (Within) | 24.027 | 0 | 0.276 |
|  | SexDyad (Between) | 0.013 | 0.987 | 0 |
|  | Task * SexDyad (Interaction) | 0.87 | 0.902 | 0.027 |
| Hdev 1/2-1Hz | Task (Within) | 6.96 | 0.098 | 0.099 |
|  | SexDyad (Between) | 0.113 | 0.955 | 0.004 |
|  | Task * SexDyad (Interaction) | 1.096 | 0.867 | 0.034 |
| Hdev 1/4-1/2Hz | Task (Within) | 0.181 | 0.805 | 0.003 |
|  | SexDyad (Between) | 1.484 | 0.704 | 0.045 |
|  | Task * SexDyad (Interaction) | 1.306 | 0.867 | 0.04 |
| Hdev 1/8-1/4Hz | Task (Within) | 2.759 | 0.29 | 0.042 |
|  | SexDyad (Between) | 0.174 | 0.955 | 0.006 |
|  | Task * SexDyad (Interaction) | 2.192 | 0.841 | 0.065 |
| Vdev 1-2Hz | Task (Within) | 10.155 | 0.031 | 0.139 |
|  | SexDyad (Between) | 2.52 | 0.488 | 0.074 |
|  | Task * SexDyad (Interaction) | 0.302 | 0.928 | 0.009 |
| Vdev 1/2-1Hz | Task (Within) | 1.503 | 0.449 | 0.023 |
|  | SexDyad (Between) | 3.004 | 0.488 | 0.087 |
|  | Task * SexDyad (Interaction) | 0.643 | 0.902 | 0.02 |
| Vdev 1/4-1/2Hz | Task (Within) | 2.568 | 0.29 | 0.039 |
|  | SexDyad (Between) | 0.721 | 0.874 | 0.022 |
|  | Task * SexDyad (Interaction) | 0.391 | 0.928 | 0.012 |
| Vdev 1/8-1/4Hz | Task (Within) | 0.042 | 0.887 | 0.001 |
|  | SexDyad (Between) | 1.274 | 0.704 | 0.039 |
|  | Task * SexDyad (Interaction) | 0.12 | 0.928 | 0.004 |

| **5.3 Task x Gender: Postural Similarity (Intersubject Euclidian Joint Distances)** | | | |  |
| --- | --- | --- | --- | --- |
| Variable | Source | F | p | np2 |
| all_mim_mean | Task (Within) | 14.467 | 0.003 | 0.187 |
|  | SexDyad (Between) | 3.489 | 0.183 | 0.1 |
|  | Task * SexDyad (Interaction) | 0.312 | 0.91 | 0.01 |
| all_mim_std | Task (Within) | 0.868 | 0.444 | 0.014 |
|  | SexDyad (Between) | 0.126 | 0.98 | 0.004 |
|  | Task * SexDyad (Interaction) | 1.337 | 0.91 | 0.041 |
| body_mim_mean | Task (Within) | 7.192 | 0.017 | 0.102 |
|  | SexDyad (Between) | 0.014 | 0.986 | 0 |
|  | Task * SexDyad (Interaction) | 0.014 | 0.986 | 0 |
| body_mim_std | Task (Within) | 8.337 | 0.013 | 0.117 |
|  | SexDyad (Between) | 0.239 | 0.98 | 0.008 |
|  | Task * SexDyad (Interaction) | 0.701 | 0.91 | 0.022 |
| upperExt_mim_mean | Task (Within) | 5.105 | 0.039 | 0.075 |
|  | SexDyad (Between) | 1.403 | 0.634 | 0.043 |
|  | Task * SexDyad (Interaction) | 0.207 | 0.91 | 0.007 |
| upperExt_mim_std | Task (Within) | 0.277 | 0.668 | 0.004 |
|  | SexDyad (Between) | 0.176 | 0.98 | 0.006 |
|  | Task * SexDyad (Interaction) | 1.731 | 0.91 | 0.052 |
| lowerExt_mim_mean | Task (Within) | 12.302 | 0.004 | 0.163 |
|  | SexDyad (Between) | 4.993 | 0.097 | 0.137 |
|  | Task * SexDyad (Interaction) | 0.398 | 0.91 | 0.012 |
| lowerExt_mim_std | Task (Within) | 0.18 | 0.672 | 0.003 |
|  | SexDyad (Between) | 0.256 | 0.98 | 0.008 |
|  | Task * SexDyad (Interaction) | 1.311 | 0.91 | 0.04 |
| head_mim_mean | Task (Within) | 9.425 | 0.011 | 0.13 |
|  | SexDyad (Between) | 1.416 | 0.634 | 0.043 |
|  | Task * SexDyad (Interaction) | 0.221 | 0.91 | 0.007 |
| head_mim_std | Task (Within) | 7.063 | 0.017 | 0.101 |
|  | SexDyad (Between) | 0.529 | 0.98 | 0.017 |
|  | Task * SexDyad (Interaction) | 0.2 | 0.91 | 0.006 |
|  |  |  |  |  |
|  | | |  |  |

| **5.4 Task x Gender: RWTLCC: Interpersonal Head Orientation** | | | | |
| --- | --- | --- | --- | --- |
| Variable | Source | F | p | np2 |
| Hdev_max_av_r | Task (Within) | 9.934 | 0.01 | 0.136 |
|  | SexDyad (Between) | 0.732 | 0.647 | 0.023 |
|  | Task * SexDyad (Interaction) | 0.9 | 0.549 | 0.028 |
| Hdev_lag_av_r_abs | Task (Within) | 1.098 | 0.597 | 0.017 |
|  | SexDyad (Between) | 0.82 | 0.647 | 0.025 |
|  | Task * SexDyad (Interaction) | 1.181 | 0.549 | 0.036 |
| Vdev_max_av_r | Task (Within) | 0.017 | 0.897 | 0 |
|  | SexDyad (Between) | 1.33 | 0.647 | 0.041 |
|  | Task * SexDyad (Interaction) | 0.076 | 0.927 | 0.002 |
| Vdev_lag_av_r_abs | Task (Within) | 0.347 | 0.744 | 0.005 |
|  | SexDyad (Between) | 0.201 | 0.818 | 0.006 |
|  | Task * SexDyad (Interaction) | 1.108 | 0.549 | 0.034 |

**Supplement 6 – Link to the Cross-Correlation Tables: Behavioral and Evaluative Data**

*https://osf.io/nafgy/?view_only=088342c029d94b489929e733fd437b15*
